# Identification, biotransformation, and neuroprotective potential of the ethanol extract of *Alpiniae oxyphyllae* fructus in neuroinflammation-related cognitive impairment

**DOI:** 10.3389/fphar.2025.1714500

**Published:** 2025-11-26

**Authors:** Zirong Yi, Xinyu Wang, Lin Wang, Renfang Yin, Dongying Qi, Huan Liu, Yuxin Chen, Yamei Xie, Yuming Ma, Mengqi Hu, Nan Zhang, Yanli Pan, Yang Liu, Guopeng Wang

**Affiliations:** 1 School of Chinese Materia Medica, Beijing University of Chinese Medicine, Beijing, China; 2 Institute of Information on Traditional Chinese Medicine China Academy of Chinese Medical Sciences, Beijing, China; 3 Zhongcai Health (Beijing) Biological Technology Development Co., Ltd., Beijing, China

**Keywords:** Alpiniae oxyphyllae fructus, sesquiterpenoids, GNPs, sequential metabolism method, blood-brain barrier permeability, anti-neuroinflammation

## Abstract

**Background:**

Neuroinflammation is a key pathological mechanism underlying various central nervous system (CNS) disorders. *Alpiniae Oxyphyllae* Fructus (AOF), the dried fruit of *Alpinia oxyphylla*, has shown therapeutic potential for these disorders. This study aimed to characterize the chemical constituents, biotransformation profiles, and neuroprotective potential of the ethanol extract of AOF in neuroinflammation-related cognitive impairment.

**Methods:**

The ethanol extract of AOF was analyzed using ultra-performance liquid chromatography coupled with high-resolution mass spectrometry (UPLC-HRMS) and Global Natural Products Social Molecular Networking (GNPS). Sequential metabolism experiments were performed to investigate its dynamic biotransformation and blood–brain barrier (BBB) permeability. Neuroprotective effects of sesquiterpenoid-enriched fractions from AOF (SE-AOF) were evaluated in a lipopolysaccharide (LPS)-induced mouse model of neuroinflammation-associated cognitive impairment.

**Results:**

A total of 108 bioactive compounds were identified from the ethanol extract of AOF. Among them, 34 compounds, mainly sesquiterpenoids, were also detected in the cerebrospinal fluid and brain tissue, indicating BBB penetration. SE-AOF significantly improved cognitive function, reduced inflammatory cytokine levels, and alleviated oxidative stress in plasma and brain tissues of LPS-treated mice.

**Conclusion:**

These findings demonstrate that the ethanol extract of AOF exerted neuroprotective effects via its bioactive sesquiterpenoids, providing insights into the therapeutic potential of natural products (NPs) against neuroinflammation-related CNS disorders.

## 1 Introduction

Neuro inflammation is increasingly recognized as a critical pathological mechanism in a wide range of central nervous system (CNS) disorders, including Alzheimer’s disease, Parkinson’s disease, and depression (Leng and Edison, 2021; Heidari et al., 2022; Salcudean et al., 2025). Persistent activation of microglia and the release of proinflammatory mediators can disrupt neuronal function, leading to cognitive impairment and neurodegeneration (Shi and Yong, 2025; Zhang et al., 2023). Although various synthetic drugs have been developed to target neuroinflammation, their efficacy remains limited and adverse effects are common. Therefore, natural products (NPs) with anti-inflammatory and neuroprotective potential are attracting growing attention as alternative or complementary therapeutic strategies.

Secondary metabolites from NPs represent a promising foundation for drug discovery, as many existing therapeutic agents are either directly or indirectly derived from these compounds (Baskiyar et al., 2022). The widespread application of ultra-performance liquid chromatography coupled with high-resolution mass spectrometry (UPLC-HRMS) has enabled the identification of thousands of complex constituents from diverse NPs (Chen T. et al., 2024; Chen S. et al., 2024; Wang et al., 2024). Nevertheless, conventional analytical workflows largely depend on literature-based manual annotation, which is labor-intensive, time-consuming, and limited in the capacity to identify unknown or novel compounds (Kong et al., 2024). To overcome these challenges, computational approaches such as molecular networking (MN) have been introduced. Global Natural Products Social Molecular Networking (GNPS), in particular, enables rapid characterization of known compounds, analogs, and potentially novel entities by constructing visualized molecular networks based on MS/MS spectral similarity. Feature-based molecular networking (FBMN), which integrates spectral feature alignment with MN, further enhances annotation accuracy, quantitative reliability, and structural isomer discrimination (Wang et al., 2016; Nothias et al., 2020). Thus, the integration of UPLC-HRMS with FBMN provides a powerful strategy for comprehensive and efficient chemical profiling of NPs.

Chemical characterization of complex constituents serves as the foundational step in pharmacological exploration, whereas systemic exposure analysis of multicomponent systems *in vivo* is critical for identifying bioactive compounds and their metabolic trajectories (Tang et al., 2023). Current *in vivo* studies often focus on metabolite profiling in biofluids such as plasma, bile, and urine. However, these approaches fail to comprehensively reveal the sequential biotransformation processes that underlie pharmacokinetics (Luo et al., 2021). The sequential metabolism strategy, which incorporates intestinal perfusion with venous sampling (IPVS) and the *in situ* closed-loop method, provides precise delineation of the gastrointestinal–hepatic–systemic circulation trajectory of com-pounds (Luo et al., 2016; Schanker et al., 1958; Liu A. et al., 2014). This methodology has been widely applied in drug metabolism research and is particularly valuable for elucidating the spatiotemporal biotransformation of both single-entity drugs and complex formulations.

*Alpiniae Oxyphyllae* Fructus (AOF), the dried fruit of *Alpinia oxyphylla*, is both a food ingredient and a traditional medicinal herb predominantly cultivated in East Asia. Historically, it has been prescribed for conditions such as frequent urination, spermatorrhea, and diarrhea. Importantly, the Compendium of Materia Medica (Ben Cao Gang Mu) documents its neuroregulatory properties, particularly in enhancing vital energy and stabilizing mental faculties. Moreover, AOF-based prescrip-tions, including Yizhi Xingnao Decoction and Yi Zhi Decoction, have demonstrated therapeutic potential against CNS disorders (Chen T. et al., 2024; Liu and Hong, 2017). Since the ability of compounds to cross the blood–brain barrier (BBB) is one of the critical determinants of their CNS pharmacological efficacy (Li et al., 2021), comprehensive identification of brain- and cerebrospinal fluid (CSF)-penetrant constituents of AOF is essential to elucidate its neuroprotective basis.

Based on this context, we hypothesized that the neuroprotective effects of AOF are mediated by specific brain-penetrant constituents that undergo unique spatiotemporal metabolic processes *in vivo*. To test this, we aimed to systematically characterize the *in vivo* biotransformation and brain distribution profile of AOF and to identify the key bioactive components responsible for its efficacy against neuroinflammation-associated cognitive impairment. The study was designed with the following objectives: (1) to delineate the dynamic metabolic trajectory of AOF constituents before and after systemic circulation; (2) to evaluate their BBB permeability; and (3) to validate the neuroprotective activity of brain-distributed compounds, particularly sesquiterpenoids, in an LPS-induced mouse model of cognitive impairment. This work provided a pharmacological foundation for the further development of AOF in the treatment of CNS disorders.

## 2 Methods

### 2.1 Reagents

AOF was purchased from Beijing Tongrentang Co., Ltd. (Beijing, China) with botanical authentication conducted by Prof. Jingjuan Wang (Beijing University of Chinese Medicine, Beijing, China), confirming its origin as desiccated mature fruits of *Alpinia oxyphylla* Miq. (family Zingiberaceae). Nootkatone (≥98%, batch No.: 23071922), Protocatechuic acid (≥98%, batch No.: 23112101), Tectochrysin (≥98%, batch No.: 24101854), and Chrysin (≥98%, batch No.: 23080810) were purchased from Beijing Bethealth People Biomedical Technology Co., Ltd. (Beijing, China). 5-Hydroxymethylfurfural (≥95%, batch No.: PS020460) and *α*-Cyperone (≥98%, batch No.: PS011728) were purchased from Chengdu Push Bio-technology Co., Ltd. (Chengdu, China). Acetonitrile (MS grade) and formic acid (MS grade) were acquired from Thermo Fisher Scientific (Waltham, MA, United States). Anhydrous ethanol (analytical grade) was acquired from Tianjin Damao Chemical Reagent Factory (Tianjin, China). Sodium chloride injection was acquired from Shijiazhuang Siyao Limited by Share Ltd. (Shijiazhuang, China). Medical adhesive was purchased from Beijing Kangpait Medical Devices Co., Ltd. (Beijing, China). Heparin sodium injection was acquired from Shanghai Pharmaceutical First Biochemical Pharmaceutical Co., Ltd. (Shanghai, China). Wahaha purified water was acquired from Hangzhou Wahaha Group Co., Ltd. (Hangzhou, China). Lipopolysaccharide (LPS) was acquired from Sigma-Aldrich (Shanghai) Trading Co., Ltd. (Shanghai, China). The NO assay kit was procured from Shanghai Biyuntian Biotechnology Co., Ltd. (Shanghai, China). The ELISA Kits for TNF-α (Cat No. 202507) and IL-6 (Cat No. 202506) were sourced from Fankewei Co., Ltd. (Shanghai, China). The SOD (Cat No. 20250619), CAT (Cat No. 20250617), and MDA (Cat No. 20250620) assay kits were purchased from Nanjing Jiancheng Bioengineering Institute (Nanjing, China). The hematoxylin and eosin staining solutions (Cat No. B1001) were purchased from BaiQianDu Bio-technology Co., Ltd. (Hunan, China). The AB-8 adsorption resin (Cat No. 25032617) was obtained from Beijing Bethealth People Biomedical Technology Co., Ltd. (Beijing, China). All additional reagents employed were of analytical grade and were readily available commercially.

### 2.2 Preparation of AOF extract

A precisely weighed 1.50 g of AOF powder was mixed with 20 times its volume of 50% ethanol and subjected to ultrasonic extraction (200 W, 40 kHz) for 30 min. The mixture was then filtered, and the residue was re-extracted under the same conditions. The two extracts were combined, and the supernatant was diluted two-fold with 50% ethanol and then filtered through a 0.22 μm microporous filter membrane. The filtrate collected was used for UPLC-HRMS analysis. The reference standards were combined in a single 5 mL volumetric flask and dissolved by ultrasonication (200 W power, 40 kHz frequency) using 3 mL methanol as the solvent. Following complete dissolution, the solution underwent microfiltration through a 0.22 μm membrane filter. The resulting filtrate was subsequently collected and prepared for UPLC-HRMS analytical characterization.

### 2.3 UPLC-HRMS analysis

Chromatographic separation was performed on a Thermo Scientific Vanquish UPLC system (Waltham, MA, United States) interfaced with a Waters ACQUITY UPLC BEH Shield RP C18 column (100 × 2.1 mm, 1.7 μm) maintained at 35 °C in a column oven. The autosampler delivered precise 5.00 μL injections. Mobile phase components comprised the following: (A) 0.1% (v/v) aqueous formic acid, and (B) Ultrapure acetonitrile. Elution was achieved at 0.30 mL/min flow rate using this gradient profile: 0.00–4.00 min: 95% A, 4.01–12.00 min: 95→80% A, 12.01–30.00 min: 60→10% A, 30.01–32.00 min: 10→95% A, 32.01–35.02 min: 95% A.

High-resolution mass spectrometric characterization was carried out on a Q-Exactive Orbitrap platform (Thermo Fisher Scientific) interfaced with a heated electrospray ionization (HESI) source operating in dual-polarity mode. Full-scan mass spectra were acquired across the *m/z* 100–1,500 range with a locked resolution of 70,000. The ionization source was configured with the following optimized parameters: capillary temperature stabilized at 320 °C, HESI probe maintained at 400 °C, and electrospray potentials set to 3.5 kV (positive mode) and 3.0 kV (negative mode). High-purity nitrogen (>99.99% purity) was employed as both sheath gas (35 arbitrary units) and auxiliary gas (10 arbitrary units), while collision-induced dissociation utilized ultrapure nitrogen (>99.99% purity) with stepped normalized collision energies (20/40/60 eV) applied in the data-dependent acquisition mode.

### 2.4 Establishment of data processing strategies

Data processing. The original UPLC-HRMS raw data were transformed into the *. mzML* format using the MSConvert tool (Holman et al., 2014). MS^1^ features and MS^2^ spectra were extracted from the raw dataset using MZmine 4.4.3 (Schmid et al., 2023). Integrated algorithms in MZmine enabled peak detection, isotope removal, peak alignment, and feature identification. For GNPS-FBMN analysis, the extracted MS^1^ peaks—with their corresponding MS^1^ and MS^2^ spectra—were exported into a *.mgf* file and a *.csv* file containing the peak quantification data.

Database construction and validation. Preliminary compound annotation was performed using Compound Discoverer 3.3 (Thermo Fisher Science) through MS/MS spectral matching against the mzCloud and mzVault databases. Xcalibur 4.2 Qual Browser facilitated manual verification by extracting ion chromatograms (mass tolerance δ ≤ 5 × 10^−6^) and cross-referencing observed fragment ions with reference standards, PubChem (https://pubchem.ncbi.nlm.nih.gov/), MassBank (https://massbank.eu/MassBank/Search), and literature-reported fragmentation patterns. Compounds that have been analyzed were compiled into a custom database, converted into *. tsv* format using the online tool (http://seriema.fcfrp.usp.br:5002/upload), and uploaded to the Network Annotation Propagation (NAP) on GNPS platform for annotation accuracy validation (Kang et al., 2018; da Silva et al., 2018).

Molecular networking construction. Processed *. mgf* and *. csv* files from MZmine were uploaded to GNPS via WinSCP. FBMN was conducted using the following criteria: a cosine score threshold of at least 0.7, a minimum requirement of 5 matched fragment ions, a topK value of 10, and the precursor mass tolerance of 0.02 Da. Networks were visualized in Cytoscape 3.10.3, where nodes represented unique compounds identified after redundancy removal and annotation, with node size indicating relative abundance. Structural similarity between compounds was assessed using cosine scores derived from MS/MS fragmentation patterns, allowing clustering of structurally related compounds; edge thickness reflected the magnitude of cosine similarity.

Based on the above analytical procedures, an in-house compound library of AOF constituents was established following manual verification. This curated library served as a reference database for subsequent identification of prototype compounds in biological samples. In addition, Compound Discoverer software was employed to facilitate the characterization of metabolites derived from *in vivo* biotransformation processes.

### 2.5 Animals

Male Sprague-Dawley (SD) rats (200–220 g) and 8-week-old male ICR mice with specific pathogen-free (SPF) status were procured from SPF Biotechnology Co., Ltd. (Beijing, China), with the qualification certificate number SCXK (Jing) 2024-0001. The animals were maintained in controlled environmental conditions (12:12 light-dark cycle, 25 °C–27 °C, 50%–70% humidity) with *ad libitum* access to food and water during the 7-day habituation phase. Prior to experimental procedures, a 12-h fasting period was implemented while maintaining water availability. All procedures involving experimental animals were conducted in strict accordance with protocols (BUCM-4–2022061502-2062) reviewed and sanctioned by the Institutional Animal Care and Use Committee (IACUC) at Beijing University of Chinese Medicine.

### 2.6 Sequential metabolism of AOF

#### 2.6.1 Stability of AOF extract in the simulated gastric juice

The simulated gastric fluid was prepared in accordance with the regulations of the Chinese Pharmacopoeia guidelines. Briefly, 16.4 mL of dilute hydrochloric acid was dissolved in 800 mL deionized water, followed by dissolving 10 g pepsin under continuous stirring. The solution was gradually diluted to 1,000 mL with additional water to achieve homogeneity. AOF extract was introduced into the simulated gastric fluid at a 1:50 ratio and incubated at 37 °C for 2 h under continuous agitation. Reactions were terminated by neutralizing the mixture to pH 6-7 using 0.1 M NaOH. For the blank group, physiological saline was used instead of AOF extract, and the remaining procedures were identical.

#### 2.6.2 IPVS surgery procedures

**FIGURE 1:**
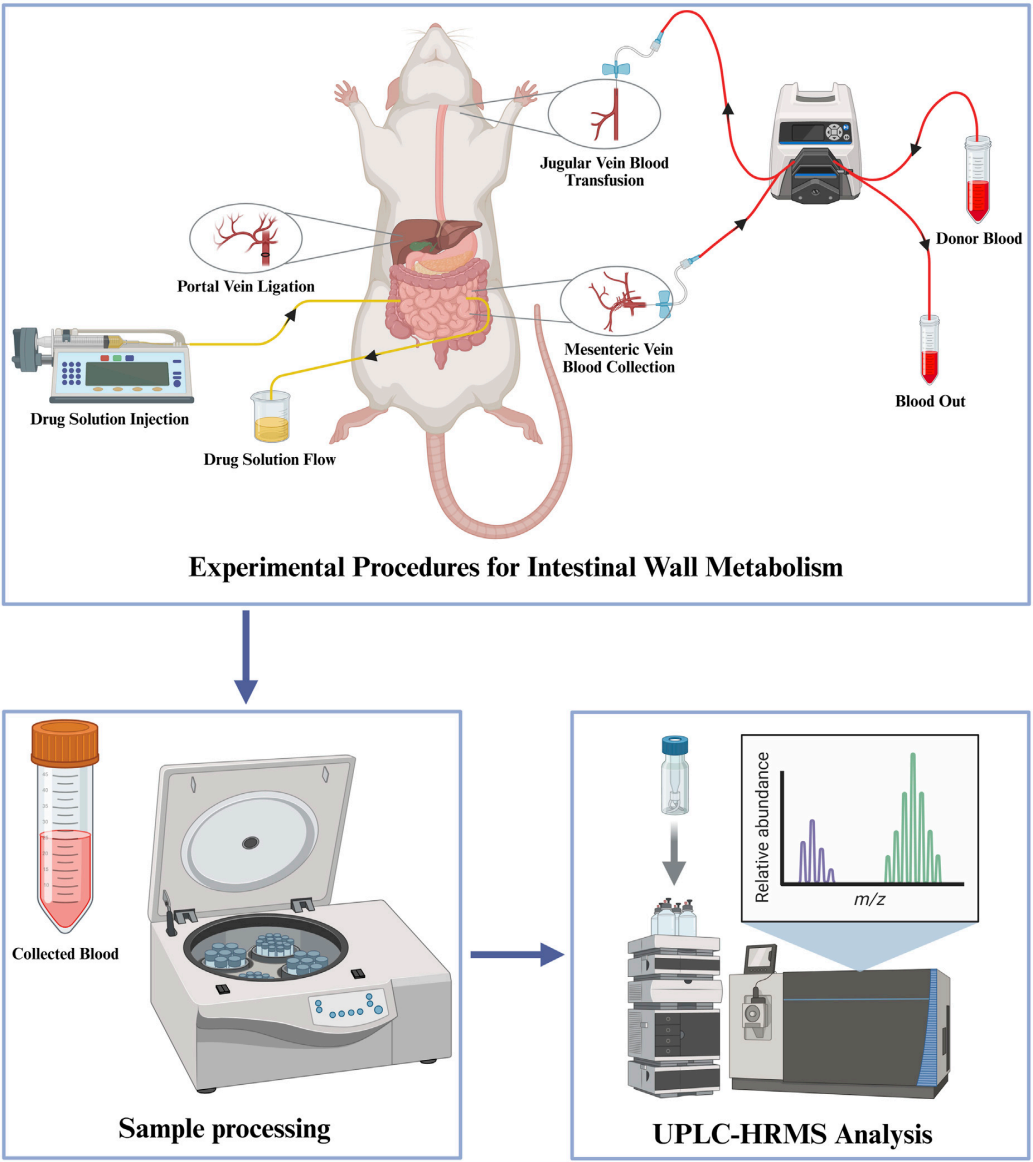
Experimental workflow for intestinal wall metabolism analysis using UPLC-HRMS.

The intestinal metabolism of AOF was investigated using IPVS by established methodologies. Six Sprague-Dawley rats (fasted for 12 h with *ad libitum* water access) were randomly assigned into two groups: five blood donor rats and one experimental subject. Donor rats were anesthetized for abdominal aorta cannulation, with collected blood in sterile centrifuge tubes and at 37 °C in a thermostatic water bath. The experimental rat underwent general anesthesia and supine positioning, followed by surgical exposure and cannulation of the external jugular vein using an indwelling needle filled with heparin sodium solution secured with medical-grade adhesive. This cannula was connected to the blood reservoir via a peristaltic pump (0.3 mL/min). The abdominal cavity was incised along the midline, and a 10-cm jejunal segment distal to the duodenum was selected as the test intestinal segment. Intestinal luminal contents were evacuated through sequential flushing with 37 °C saline until effluent clarification, followed by air insufflation to eliminate residual fluid. AOF extract (1 g/mL) was perfused via a syringe pump at 0.2 mL/min, with distal effluent discarded. Hepatic portal vein ligation isolated intestinal metabolism while an indwelling needle filled with heparin sodium solution was inserted into the mesenteric vein, connected to a peristaltic pump for continuous blood collection (0.3 mL/min over 120 min). Exposed intestinal tissue was protected with saline-moistened gauze and maintained at 37 °C using a heat lamp. The schematic diagram of the experimental procedure for Intestinal wall metabolism is shown in Figure 1.

For the hepatic metabolism experiment, the protocol was modified by cannulating the femoral vein to collect blood while maintaining hepatic portal circulation patency. Mesenteric venous samples represented intestinal-specific metabolism, whereas femoral venous samples contained combined hepatic and intestinal metabolites.

#### 2.6.3 *In situ* closed-loop method

Following anesthesia induction, a colonic segment approximately 8 cm distal to the cecum was isolated as the experimental intestinal compartment. The AOF extract (1 g/mL) was infused into the colonic lumen via syringe. Upon complete perfusion of the test solution to the distal end of the isolated segment, the terminal portion was ligated with sterile surgical silk. The infusion syringe was then carefully disengaged, followed by proximal ligation to create a closed-loop system. Subsequent procedures were executed following the established intestinal metabolism.

#### 2.6.4 Oral drug administration

Rats were randomly divided into 8 groups, with 3 animals in each group. Treatment groups received AOF extract (1 g/mL, 2 mL per rat) via oral gavage, whereas control groups were given isovolumetric saline. After being anesthetized, terminal blood samples were obtained via abdominal aortic puncture at 5, 15, 30, 45, 60, 90, and 120 min post-dosing using heparinized syringes. Blood obtained via abdominal aorta sampling reflects the systemic equilibrium established through integrated biotransformation processes by different organs and microbial flora (Liu et al., 2023).

Following abdominal aorta blood sampling, cardiac perfusion was systematically performed prior to the CSF and brain tissue (BT) collection to prevent blood contamination (Wu et al., 2021). The thoracic cavity was opened through thoracotomy, and the pericardium was incised to expose the heart. A perfusion cannula was inserted into the left ventricle and secured, followed by the creation of an outflow aperture at the right atrial appendage. Prechilled saline solution (4 °C) was rapidly infused until the liver exhibited clay-like pallor, pulmonary and ocular tissues turned pale, and the perfusate became clear. After completing cardiac perfusion, the rat was positioned prone on a surgical platform with its head fixed at a 135° body-to-head angulation. The occipital region was shaved and disinfected, and a 1.5 cm cruciform incision was made over the foramen magnum. Through blunt dissection of the trapezius and semispinalis capitis muscles, the atlanto-occipital membrane and foramen magnum were exposed. A custom CSF aspiration device (blood-collection needle attached to a heparinized 1 mL syringe) was inserted posterolaterally through the foramen magnum toward the cisterna magna. Gentle aspiration yielded approximately 100 μL of clear CSF. Subsequently, the rat was decapitated, whose brain was carefully *en bloc* from the cranial cavity, showing uniform ivory-white coloration without residual blood. All blood samples were pooled to form composite specimens, while CSF and BT were respectively aggregated into unified biological replicates for subsequent processing.

#### 2.6.5 Biological samples pretreatment

Blood samples: After collection, blood samples were centrifuged at 1,164 *g* for 10 min at 4 °C. The clarified supernatant was homogenized with an equivalent volume of MS-grade acetonitrile (1:1 v/v), vortexed for 2 min to ensure complete protein precipitation. The mixture was then centrifuged again at 9,503 × g for 10 min at 4 °C. The resulting supernatant was dried under nitrogen gas, and then reconstituted with 300 µL of 50% methanol. After centrifugation (9,503 × g, 10 min, 4 °C), the resulting supernatant was ready for immediate UPLC-HRMS analysis as previously described in Section 2.3.

CSF samples: An equivalent volume of MS-grade acetonitrile was added to the CSF samples, mixed, and vortexed for 2 min. Subsequent procedures are identical to the processing steps described for the blood samples.

BT samples: The weight of the BT was precision-measured, and then 3 times the volume of physiological saline was added for homogenization for 3 min. Subsequently, 5 times the volume of pure acetonitrile was added, mixed, and vortexed for 2 min. Subsequent procedures are identical to the processing steps described for the blood samples.

### 2.7 Investigation of the effects of SE-AOF on the LPS-induced mice

#### 2.7.1 Preparation of SE-AOF extract

An AB-8 macroporous resin column was employed to enrich sesquiterpenoids from AOF extracts (SE-AOF). Nootkatone and *α*-cyperone were used as reference standards, and the enriched fraction was quantified by HPLC. The optimized purification conditions were as follows: flow rate 0.5 mL/min, a loading of 3 g crude drug per g resin, and gradient ethanol elution. Impurities were removed by sequential elution with water, 30% and 50% ethanol, and the SE-AOF fraction was collected with 95% ethanol. The 95% ethanol eluate was concentrated to dryness under reduced pressure, accurately weighed, and reconstituted in 0.5% carboxymethyl cellulose sodium (CMC-Na) to 100 mg/mL for oral administration.

#### 2.7.2 Groups and treatment

Forty-eight mice were randomly assigned to six groups (n = 8): control, model, donepezil (1.3 mg/kg/day), and SE-AOF low-, medium-, or high-dose groups (25, 50, and 100 mg/kg/day, respectively). Neuroinflammation-associated cognitive impairment was induced by daily intraperitoneal injections of LPS (500 μg/kg) for 4 weeks in all groups except the control group. Drug-treated groups received oral gavage of the assigned intervention 3 h after each LPS injection. SE-AOF doses were determined based on clinical equivalence and enrichment of bioactive sesquiterpenoids, with 50 mg/kg/day as the medium dose and the low and high doses set at half and double this value, respectively. Donepezil was selected as the positive control based on its well-established efficacy in improving cognitive impairment in mice (Zhou et al., 2019; Xu et al., 2022; Kim et al., 2021). Control mice were injected with saline and gavaged with the 0.5% CMC-Na vehicle, serving as the naïve control. Model mice received LPS injections and were gavaged with the 0.5% CMC-Na vehicle to represent the disease model without pharmacological intervention. All oral administrations used 0.5% CMC-Na to ensure uniform dispersion of SE-AOF and comparable gavage conditions across groups. LPS was dissolved in sterile saline for intraperitoneal injection. The experimental design is illustrated in Figure 8A.

#### 2.7.3 Y-maze test

Spatial learning and memory were assessed using spontaneous alternation and novel-arm exploration. To minimize potential olfactory interference between animals, the Y-maze apparatus was thoroughly wiped with 70% ethanol after each mouse trial, followed by a 3-min interval to allow complete evaporation of ethanol before the next test.

Spontaneous alternation: Mice were placed in a Y-maze (30 cm × 6 cm × 20 cm) and allowed to explore freely for 5 min (Abdulbasit et al., 2018). A correct alternation was defined as consecutive entries into all three arms; otherwise, the event was considered incorrect. Trajectories were recorded and analyzed using EthoVision XT (Noldus). The alternation rate was calculated as:

Spontaneous alternation (%) = number of alternations ÷ (total arm entries −2) × 100%.

Novel-arm exploration: During Phase I, one arm was blocked and designated as the “novel arm” and mice explored the other two arms for 5 min. Four hours later (Phase II), the barrier was removed and mice explored all three arms for 5 min (Zborowski et al., 2024). Movement was recorded by EthoVision XT. Indices included:

- Percentage of entries into the novel arm = novel-arm entries ÷ total entries × 100%;
- Percentage of time in the novel arm = cumulative time in the novel arm ÷ total time × 100%.

#### 2.7.4 Open field test

Exploratory activity, locomotion, and anxiety-like behavior were assessed in a 50 cm × 50 cm × 40 cm arena. The apparatus was cleaned with 70% ethanol and allowed to dry for 3 min between animals to eliminate potential odor cues. Mice were placed in the center and allowed to explore for 10 min (Chen W. et al., 2023). Total distance traveled, number of center entries, and time spent in the center were analyzed with EthoVision XT.

#### 2.7.5 Tissue preparation

After behavioral testing, mice were anesthetized, secured in the supine position, and transcardially perfused with ice-cold saline. Brains were removed and weighed. The left hemisphere was stored at −80 °C for biochemical assays, and the right hemisphere was fixed in 4% paraformaldehyde for histology and immunohistochemistry.

#### 2.7.6 Histological assessment by H&E staining

Hematoxylin-eosin (H&E) staining was performed to evaluate hippocampal morphology (Fu et al., 2025). Fixed brains underwent graded ethanol dehydration, clearing, and paraffin embedding. Sections (4 μm; RM2016, China) were stained using standard H&E procedures. Histological evaluation was conducted by observers blinded to group allocation.

#### 2.7.7 Immunohistochemical assessment of microglial activation

Microglial number and morphology in the hippocampus and cortex were examined by IBA-1 immunohistochemistry (Norden et al., 2016). After fixation, dehydration, embedding, and sectioning, sections were incubated with an anti-IBA-1 primary antibody followed by the appropriate secondary antibody. Image acquisition and quantification were performed under blinded conditions.

#### 2.7.8 Inflammatory markers

Plasma levels of tumor necrosis factor-*α* (TNF-α) and interleukin-6 (IL-6), and brain levels of nitric oxide (NO), TNF-α, and IL-6, were measured according to the manufacturers’ instructions. Given the study focus on neuroinflammation, inclusion of plasma assays enabled assessment of peripheral inflammatory status and its potential impact on central inflammation, allowing a more comprehensive evaluation of the intervention’s regulatory effects.

#### 2.7.9 Oxidative stress markers

Activities of superoxide dismutase (SOD) and catalase (CAT), and levels of malondialdehyde (MDA) in the plasma and brain, were determined according to the kit protocols. Because oxidative stress is tightly linked to inflammatory processes, these measurements provided complementary systemic and central readouts that could be integrated with inflammatory markers to characterize the overall effects of the interventions.

### 2.8 Statistical analysis

All data are represented as mean ± standard error of the mean (SEM). Statistical analyses were performed using GraphPad Prism 9.0 (GraphPad Software, United States). Data normality and homogeneity of variance were first evaluated using the Shapiro–Wilk and Levene’s tests, respectively. Differences among multiple groups were analyzed by one-way analysis of variance (ANOVA) followed by Tukey’s *post hoc* test for pairwise comparisons. Nonparametric tests were applied when data did not meet parametric assumptions. A *p* value <0.05 was considered statistically significant.

## 3 Results and discussion

### 3.1 Compound identification in AOF extract

**FIGURE 2:**
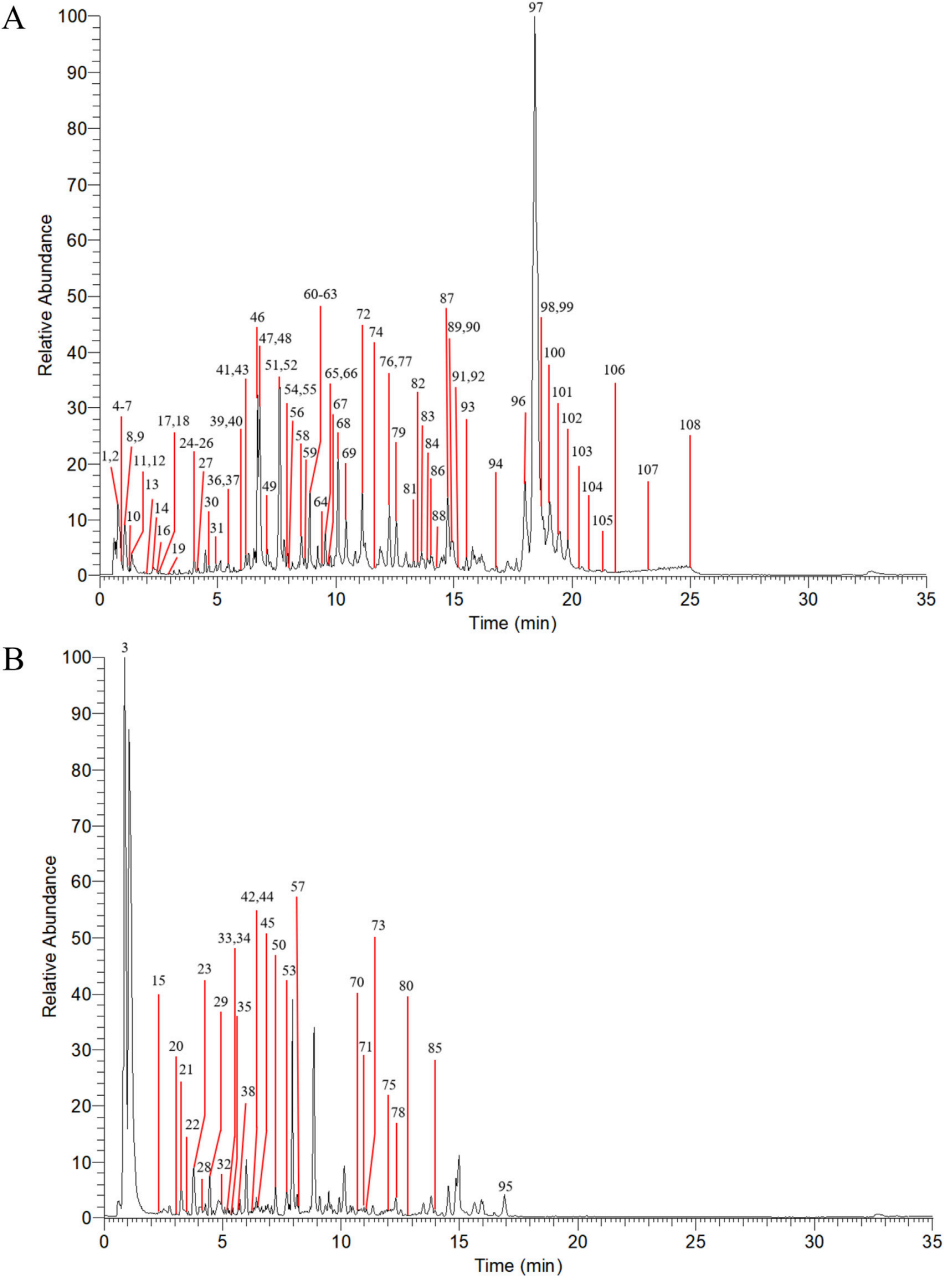
Base peak intensity (BPI) chromatograms for AOF in positive ion mode **(A)** and negative ion mode **(B)**.

**FIGURE 3:**
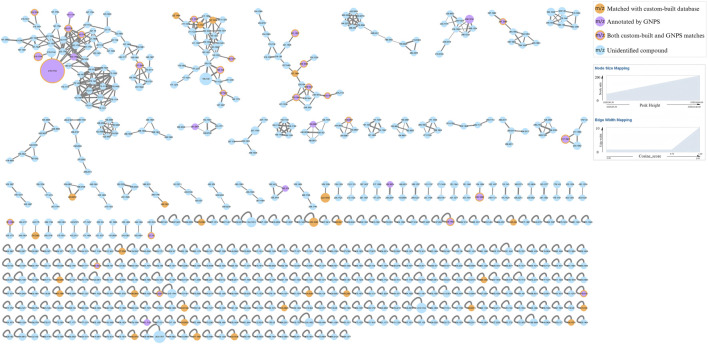
The Feature-based molecular networking diagram of AOF in positive ion mode.

The MS data of AOF extract were acquired in both positive and negative ion modes using UPLC-HRMS, and the resulting Base Peak Ion (BPI) chromatograms are shown in Figure 2. The FBMN analysis revealed 666 nodes in positive ion mode, with 67 clusters containing ≥2 nodes in Figure 3. In negative ion mode, the FBMN comprised 752 nodes and 66 clusters with ≥2 nodes in Supplementary Figure S1.

A curated database of 89 AOF compounds was established, comprising detailed physicochemical and spectral information; 33 of these were further validated by NAP annotation, supporting the accuracy of structural identification. During molecular networking analysis, GNPS annotation identified 26 compounds, 7 of which overlapped with pre-existing entries in the custom-built database. Collectively, these efforts resulted in the identification of 108 distinct compounds. The identified compounds included terpenoids, organic acids, flavonoids, esters, aldehydes, and other classes. Detailed compound identification results with corresponding information are summarized in Supplementary Table S1.

#### 3.1.1 Identification of terpenoid compounds

Terpenoids were identified as the most abundant constituents in AOF. A total of 43 terpenoid components were characterized, including 41 sesquiterpenoids and 2 monoterpenoids. Sesquiterpenoids, which are composed of three isoprene units and contain fifteen carbon atoms, represent a class of compounds that are generally regarded as both characteristic components and primary active constituents in AOF (Wang et al., 2014).

**FIGURE 4:**
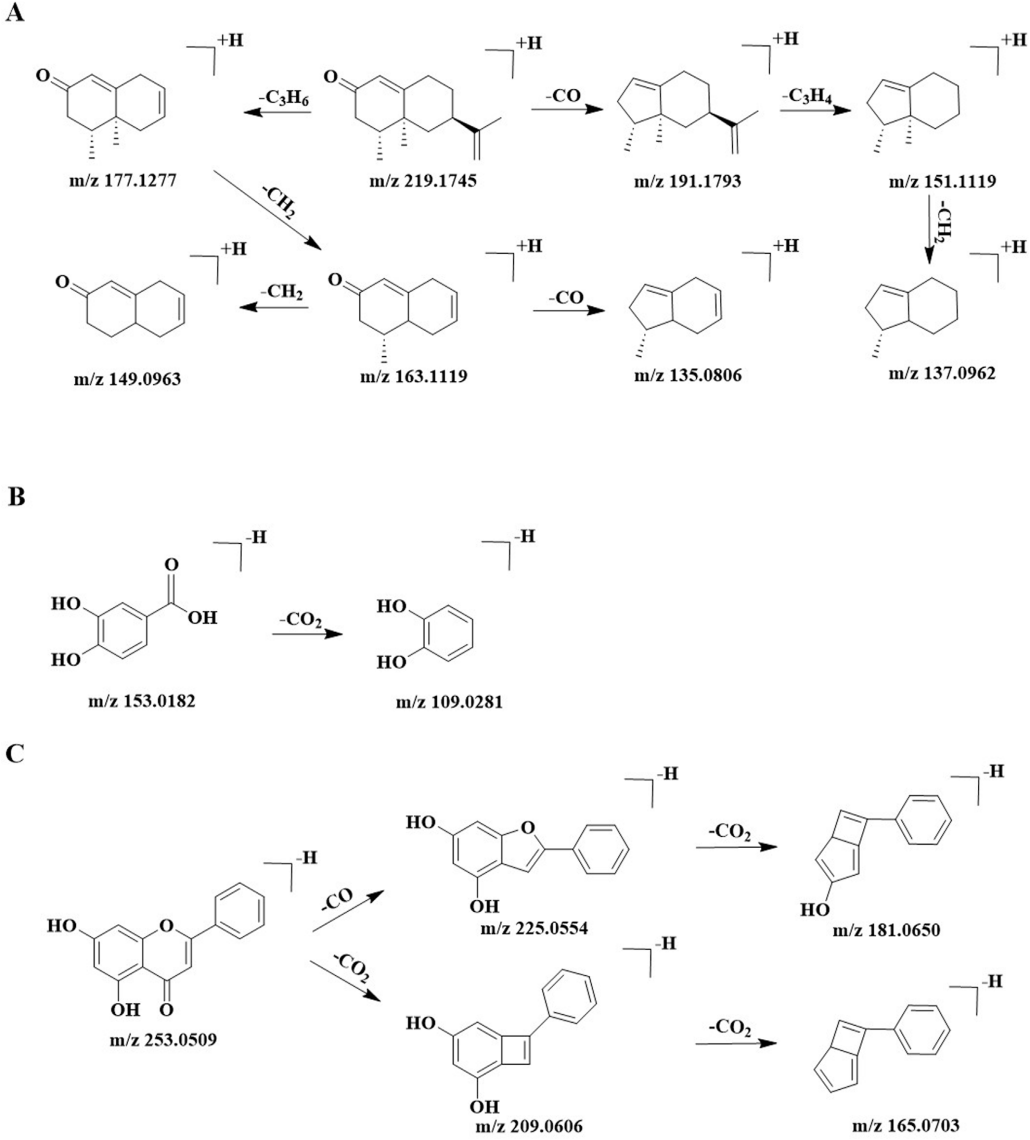
Possible fragment pathways of Nootkatone **(A)**, Protocatechuic acid **(B)**, and Chrysin **(C)** from AOF.

Taking Peak 97 (Nootkatone) as an exemplar, its protonated molecular ion [M + H]^+^ was unambiguously identified at *m/z* 219.1743 in positive ionization mode. Qual Browser software determined its precise molecular formula as C_15_H_22_O with a mass error of −0.237 ppm. Diagnostic fragment ions in MS/MS spectra were identified at *m/z* 191.1793, 177.1277, 163.1119, 151.1119, 149.0963, 137.0962, and 135.0806. The precursor ion underwent neutral loss of a carbonyl group (CO, 28 Da) to generate the fragment at *m/z* 191.1793, while sequential elimination of a propyl radical (C_3_H_6_, 42 Da) produced the ion at *m/z* 177.1277. Subsequent cleavage of the *m/z* 191.1793 species through allylic dehydrogenation (C_3_H_4_, 40 Da) yielded the diagnostic fragment at *m/z* 151.1119. Further methylene group (CH_2_, 14 Da) abstraction from *m/z* 151.1119 resulted in *m/z* 137.0962. Parallel frag-mentation of *m/z* 177.1277 demonstrated consecutive demethylation events through two CH_2_ losses (14 Da × 2), producing *m/z* 163.1119 and 149.0963. Notably, the *m/z* 135.0806 fragment emerged via retro-Diels-Alder-type elimination of CO (28 Da) from *m/z* 163.1119. Structural confirmation as Nootkatone was achieved through comparison with an authentic reference standard and literature data (Zhang et al., 2022). The proposed fragmentation pathway is illustrated in Figure 4A.

**FIGURE 5:**
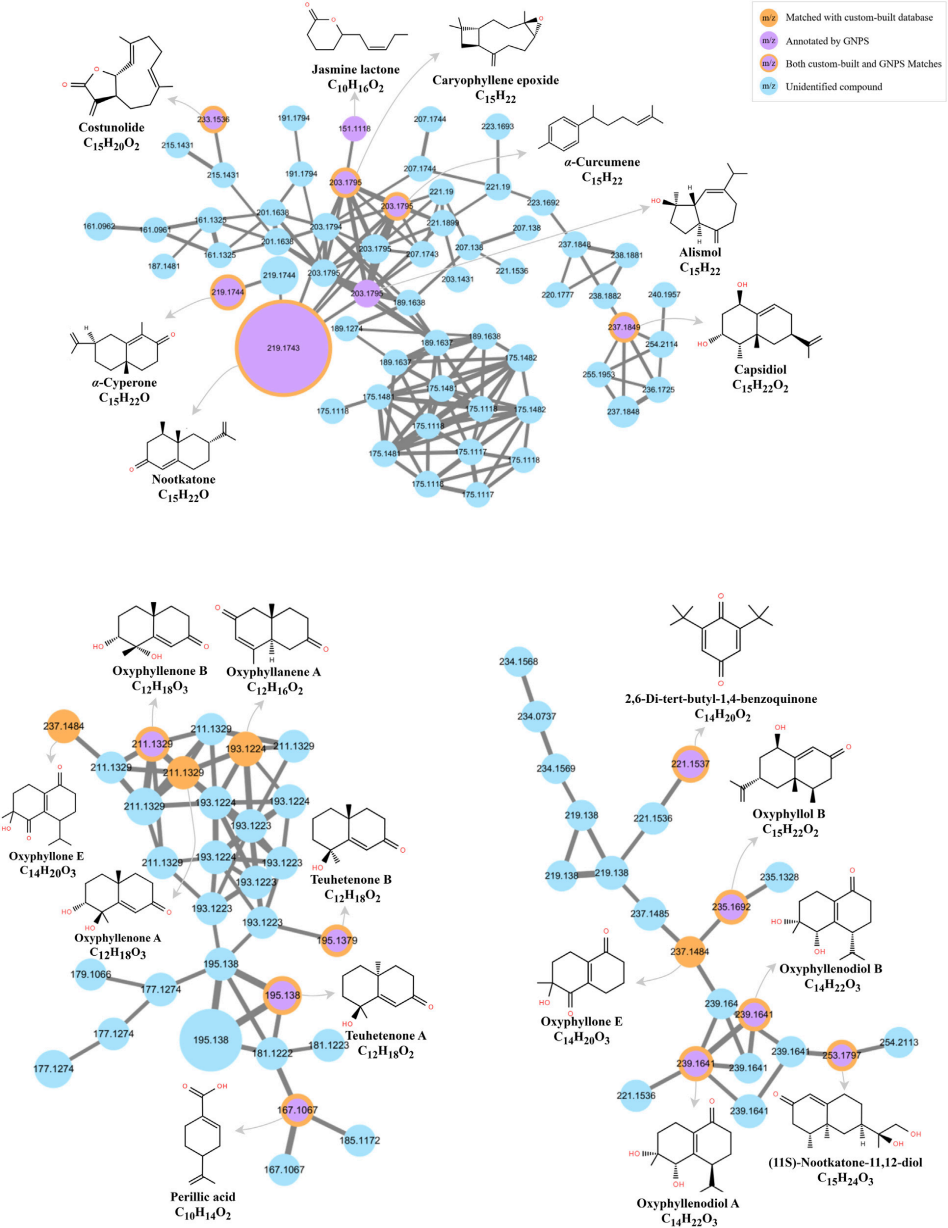
The feature-based molecular network revealing dominant sesquiterpenoid clusters in the AOF extract.

These compounds are mainly represented in the molecular network obtained in positive ion mode, and owing to structural similarities, they are predominantly clustered within the three largest clusters. A detailed molecular network is shown in Figure 5.

#### 3.1.2 Identification of organic acid compounds

A total of 26 organic acid compounds, including Protocatechuic acid and Jasmonic acid, were identified in this study. These components are prone to neutral losses of CO_2_, CH_2_, and CO during collision-induced dissociation in mass spectrometry (Darshan et al., 2014). Taking Peak 21 (Protocatechuic acid) as a representative example, its deprotonated molecular ion ([M-H]^-^) was definitively observed at *m/z* 153.0183 in negative ionization mode. Qual Browser software assigned the precise molecular formula as C_7_H_6_O_4_ with a mass error of 0.162 ppm. Collision-induced dissociation analysis revealed two diagnostic product ions at *m/z* 153.0183 and 109.0281. The characteristic fragment at *m/z* 109.0281 originated from decarboxylation of the precursor ion, corresponding to neutral elimination of carbon dioxide (CO_2_, 44 Da). Structural confirmation as protocatechuic acid was achieved by comparison with an authentic reference standard and literature data (Chen Y. et al., 2023). The proposed fragmentation pathway is illustrated in Figure 4B.

These compounds primarily appear in the molecular network obtained in negative ion mode. However, aside from all possessing a carboxyl group (-COOH), their other structural features differ substantially, resulting in markedly distinct MS/MS spectra and subsequent cluster separation (Qin et al., 2022). Therefore, their molecular network diagrams are not individually represented.

#### 3.1.3 Identification of flavonoid compounds

In this study, a total of 6 flavonoids were identified, including Procyanidin B1, (+)-Catechin, Sinensetin, Nobiletin, Chrysin, and Tectochrysin. Taking Peak 95 (Chrysin) as an example, its quasi-molecular ion peak was observed at *m/z* 253.0505 ([M-H]^-^) in negative ion mode. The exact molecular formula determined by Qual Browser software was C_15_H_10_O_4_ (error: 0.975 ppm). The major MS/MS product ions were identified at *m/z* 225.0554, 209.0606, 181.0650, and 165.0703. The diagnostic ion at *m/z* 225.0554 was generated through the loss of 28 Da (CO) from the deprotonated molecular ion, while the ion at *m/z* 209.0606 resulted from the elimination of 44 Da (CO_2_). Subsequently, both product ions underwent further fragmentation: the ion at *m/z* 225.0554 underwent a neutral loss of a carbon dioxide molecule (CO_2_, 44 Da), generating a fragment ion at *m/z* 181.0650; similarly, the ion at *m/z* 209.0606 eliminated a carbon monoxide molecule (CO, 28 Da), yielding a fragment ion at *m/z* 165.0703. The compound was conclusively identified as Chrysin through comparison with an authentic reference standard and literature data (Yan, 2009). The proposed fragmentation pathways of the product ions are illustrated in Figure 4C.

These compounds appear in molecular networks obtained in both positive and negative ion modes. Although they share the same flavonoid nucleus, variations in substituent positions, stereochemistry, or functional group modifications (such as hydroxylation and methoxylation) could lead to significantly different MS/MS spectra and subsequent cluster separation. Therefore, their molecular network diagrams are not individually represented.

#### 3.1.4 Identification of other compound types

In addition to the chemical classes mentioned above, esters, aldehydes, amino acids, alkaloids, and other compounds were also identified from the AOF extract. Fragmentation pathways vary among these classes, and most of these compounds appear as single and isolated nodes in the molecular network; therefore, they are not shown individually here.

### 3.2 Compound identification in different biological samples

#### 3.2.1 Stability in the simulated gastric juice

The *in vivo* stability of a drug is a critical factor in quality assessment, and thus all potential factors affecting its stability must be comprehensively evaluated prior to systemic circulation (Mathieu et al., 2015). For oral administration, the acidic gastric environment and the presence of pepsin pose significant challenges to compound stability. It is worth mentioning that 95 peaks were identified in post-incubation samples, demonstrating that AOF remained remarkably stable during gastric digestion. Comprehensive details for all components are provided in Supplementary Table S2.

#### 3.2.2 Identification of absorbed prototypes in blood samples

Based on the sequential metabolism theory, this study systematically elucidated the *in vivo* biotransformation process of AOF by IPVS and *in situ* closed-loop method. The BPI chromatograms of various blood sample groups treated with AOF are represented in Supplementary Figure S2. A total of 63 prototype compounds were identified in the blood samples, consistent with the molecular characteristics of parent constituents detected in the AOF extract. Detailed information is provided in Supplementary Table S2. Compared with the simulated gastric juice (SGJ) group, the result suggests that intestinal wall enzymes, gut microbiota, and hepatic metabolic enzymes may influence the absorption and metabolism processes of certain AOF-derived chemical constituents.

Specifically, 48 components were identified in the MB-W group (mesenteric blood from intestinal wall metabolism) and 47 components in the MB-F group (mesenteric blood from intestinal flora metabolism), with 42 components common to both groups. This overlap indicates that these shared components remain essentially stable in the gastrointestinal tract, either not being metabolized or undergoing partial metabolism, while maintaining the capability to be absorbed through the intestinal wall into the bloodstream. A previous study employing a rat single-pass intestinal perfusion model demonstrated favorable absorption of Nootkatone across duodenal, jejunal, and ileal segments, primarily through passive diffusion mechanisms, with maintained stability under varying pH conditions (Wu et al., 2016). In this study, the observed stability of multiple sesquiterpenoid compounds, exemplified by Nootkatone (Peak 97), within the gastrointestinal environment aligns with the earlier finding. Six components were uniquely detected in the MB-W group but absent from the MB-F group. This phenomenon is consistent with possible catabolic degradation by colonic microbiota, which are known to mediate extensive metabolic transformation of dietary and phytochemical constituents (Rowland et al., 2018; Zhao et al., 2023). Further studies will be conducted to clarify whether intestinal microbiota contribute to these metabolic differences.

Due to surgical processing, the FVB group (femoral vein blood from hepatic metabolism) reflects combined hepatic and intestinal metabolic influences. To isolate liver-specific metabolism, comparative analysis between the MB-W and FVB groups was conducted. 34 components were detected in the FVB group, representing a reduction of 14 components compared to the MB-W group. This disparity could potentially be attributed to hepatic metabolic enzyme activity mediating the biotransformation of these components. Notably, several organic acids and flavonoids—including Protocatechuic acid (Peak 21), Chrysin (Peak 95), and Tectochrysin (Peak 101)—were undetectable across all three groups. A study employing metabolite analysis of Protocatechuic acid *in vivo* has demonstrated that its hydroxyl groups undergo rapid Phase II conjugation with sulfate and glucuronic acid post-absorption, thereby substantially diminishing the circulating levels of the parent compound to below the detection threshold, which precludes its reliable identification (Chen et al., 2017). Moreover, investigations into Chrysin and Tectochrysin have further demonstrated their characteristically low oral bioavailability, attributed to hydroxyl groups within their chemical structures that predispose these flavonoids to rapid Phase II conjugation reactions, while significant amounts of parent compounds have been detected in fecal samples (Walle et al., 2001; Qin et al., 2014).

Furthermore, 59 compounds were identified in the AA group (oral administration with time-phased abdominal aorta blood sampling). Comparative analysis of the four experimental groups revealed that AA group exhibited detection of a greater number of compounds. Three plausible explanations are proposed: (i) site-specific absorption: Upon oral administration, some compounds may undergo rapid absorption in the stomach or proximal small intestine (e.g., duodenum), regions not accessible via jejunum/colon administration; (ii) metabolic-regeneration mechanisms: Partial metabolism or transformation of some compounds may occur locally (e.g., intestinal wall or liver), yet their parent forms could be regenerated through enterohepatic circulation or reabsorption pathways (Ibarra et al., 2021; Roberts et al., 2002); (iii) lymphatic absorption: Highly lipophilic compounds may bypass hepatic first-pass metabolism via lymphatic transport, directly entering systemic circulation (Tso et al., 2025; Trevaskis et al., 2008). In future work, we will conduct in-depth studies to further verify these hypotheses and elucidate the underlying mechanisms.

#### 3.2.3 Identification of BBB-penetrating compounds

The unique physiological structure of the BBB maintains cerebral homeostasis by blocking toxic substances, yet simultaneously hinders the delivery of hydrophilic drugs and macromolecular therapeutics to the CNS, thereby posing a major obstacle to drug therapy for CNS disorders (Wu et al., 2023). Consequently, overcoming the BBB remains a critical challenge for developing effective pharmacological interventions targeting the CNS.

Based on the identified chemical and blood-absorbed components of AOF, this study further characterized BBB-penetrating constituents of AOF by integrating secondary fragment ion analysis with Xcalibur 4.2 software. Qualitative analysis of AOF-derived components in rat CSF and BT samples was performed. The BPI chromatograms under positive and negative ion modes are shown in Supplementary Figure S1. After excluding endogenous component interference through comparison with blank controls, 34 BBB-permeable AOF constituents were identified: 30 in CSF and 29 in BT. Detailed information is shown in Supplementary Table S2. Notably, 26 sesquiterpenoids were detected among these constituents, whose low molecular weight and high lipophilicity conferred significant advantages in BBB penetration.

The differential composition analysis between CSF and brain tissue revealed that identified components could be categorized into three groups: (i) detected in both CSF and brain tissue: These compounds are hypothesized to be absorbed into the bloodstream in their prototype form following gastrointestinal digestion and metabolism by hepatic enzymes and intestinal wall enzymes. Subsequently, they traverse both the BBB and the blood-cerebrospinal fluid barrier (BCSFB) to enter brain tissue and CSF. Most BBB-permeable compounds fall into this category; (ii) detected in brain tissue but not in CSF: This discrepancy may arise from distinct local transport mechanisms. The BBB and BCSFB represent two distinct physiological barriers (Lin, 2008). Certain components may cross the BBB into brain tissue but fail to enter CSF due to restrictions imposed by the BCSFB or the CSF-brain barrier. Additionally, rapid clearance during CSF circulation may prevent their detection in CSF, whereas slower clearance in brain tissue allows their accumulation. Furthermore, localized metabolic processes in brain tissue may convert these components into other metabolites, rendering them undetectable in CSF. Conversely, reduced metabolic activity in brain tissue may enable their stable retention and detection, as observed for Peak 82, 86, 92, and 93; (iii) detected in CSF but not in brain tissue: These compounds are likely transported into CSF via the BCSFB but are restricted from entering brain tissue by the BBB or CSF-brain barrier, as exemplified by Peak 40, 41, 84, and 87.

In modern pharmacological research, numerous studies have utilized solvent-based extracts of AOF (e.g., aqueous extract, ethanol extract) (Koo et al., 2004; Wang et al., 2018) and its monomeric compounds (e.g., Nootkatone, 5-Hydroxymethylfurfural) (He et al., 2018; Liu Y. et al., 2014) to investigate their therapeutic effects on CNS diseases. These studies have demonstrated their activities in counteracting neuroinflammation, alleviating oxidative stress, and enhancing synaptic plasticity. The present study provides crucial experimental proof supporting the potential of AOF for the prevention and treatment of CNS disorders, addressing the previous research gap in tracing the origins of AOF-derived bioactive components with CNS-specific effects. Furthermore, this work pioneers novel research avenues for future investigations into AOF’s mechanisms and applications within the CNS, thereby advancing its prospects in neurological therapeutics.

#### 3.2.4 Identification of metabolites across diverse biological samples

Within the complex environment of the body, drug components, due to their structural diversity, may undergo biotransformation upon entering the body, generating multiple metabolites through diverse metabolic reactions. These metabolites may exhibit pharmacological activities similar to, greater than, lesser than, or different from those of the parent compounds. Investigating metabolites enables a more comprehensive understanding of true therapeutic efficacy of the drug *in vivo*, thereby avoiding the oversimplification of predicting *in vivo* effects based solely on the *in vitro* activity of the parent drug.

**FIGURE 6:**
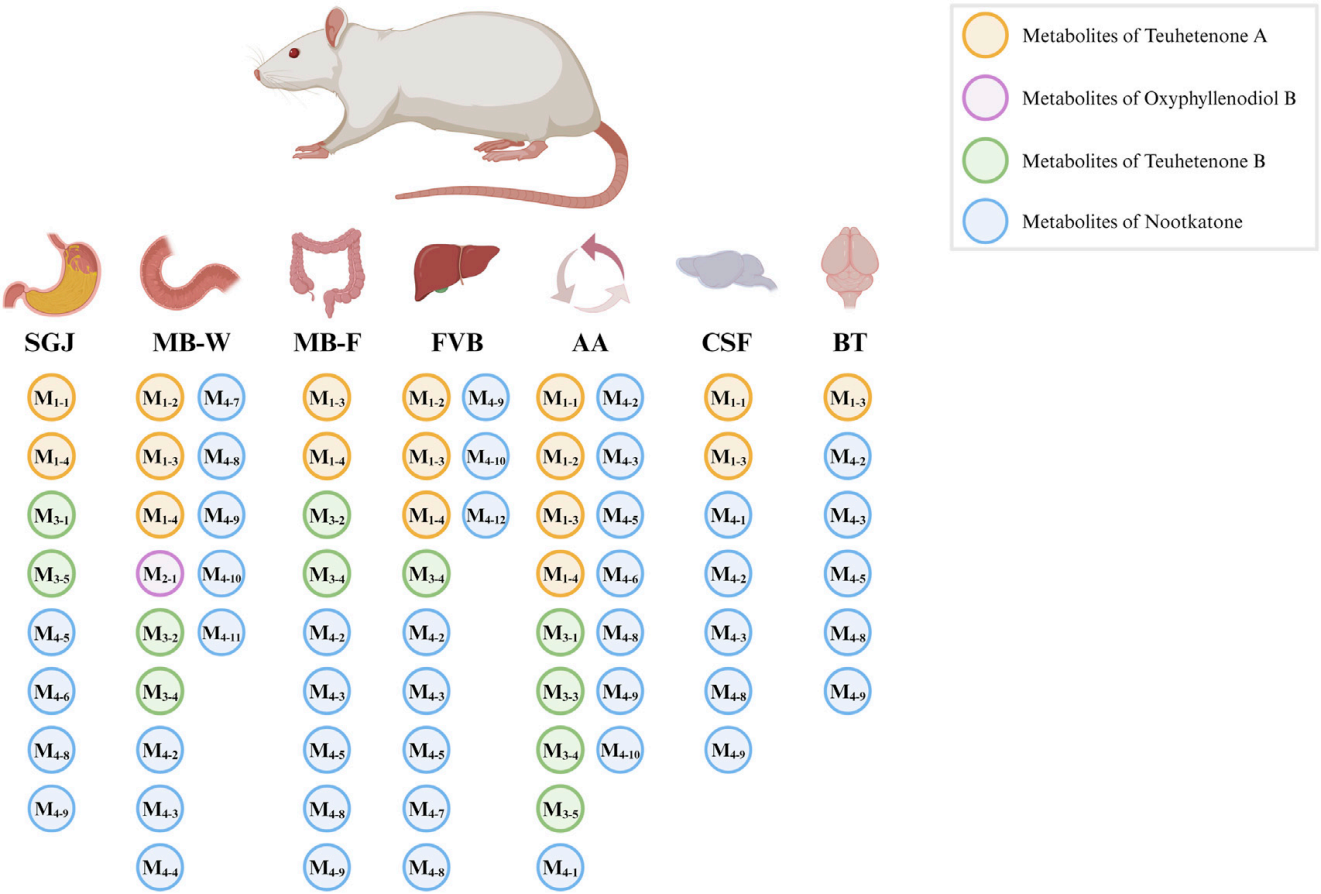
Multicompartment metabolic profiling of AOF: metabolite distribution from gastrointestinal processing to cerebral tissue translocation. Note: SGJ, simulated gastric juice; MB-W, mesenteric blood from intestinal wall metabolism group; MB-F, mesenteric blood from intestinal flora metabolism group; FVB, femoral venous blood from hepatic metabolism; AA, abdominal aorta; CSF, cerebrospinal fluid; BT, brain tissue.

**FIGURE 7:**
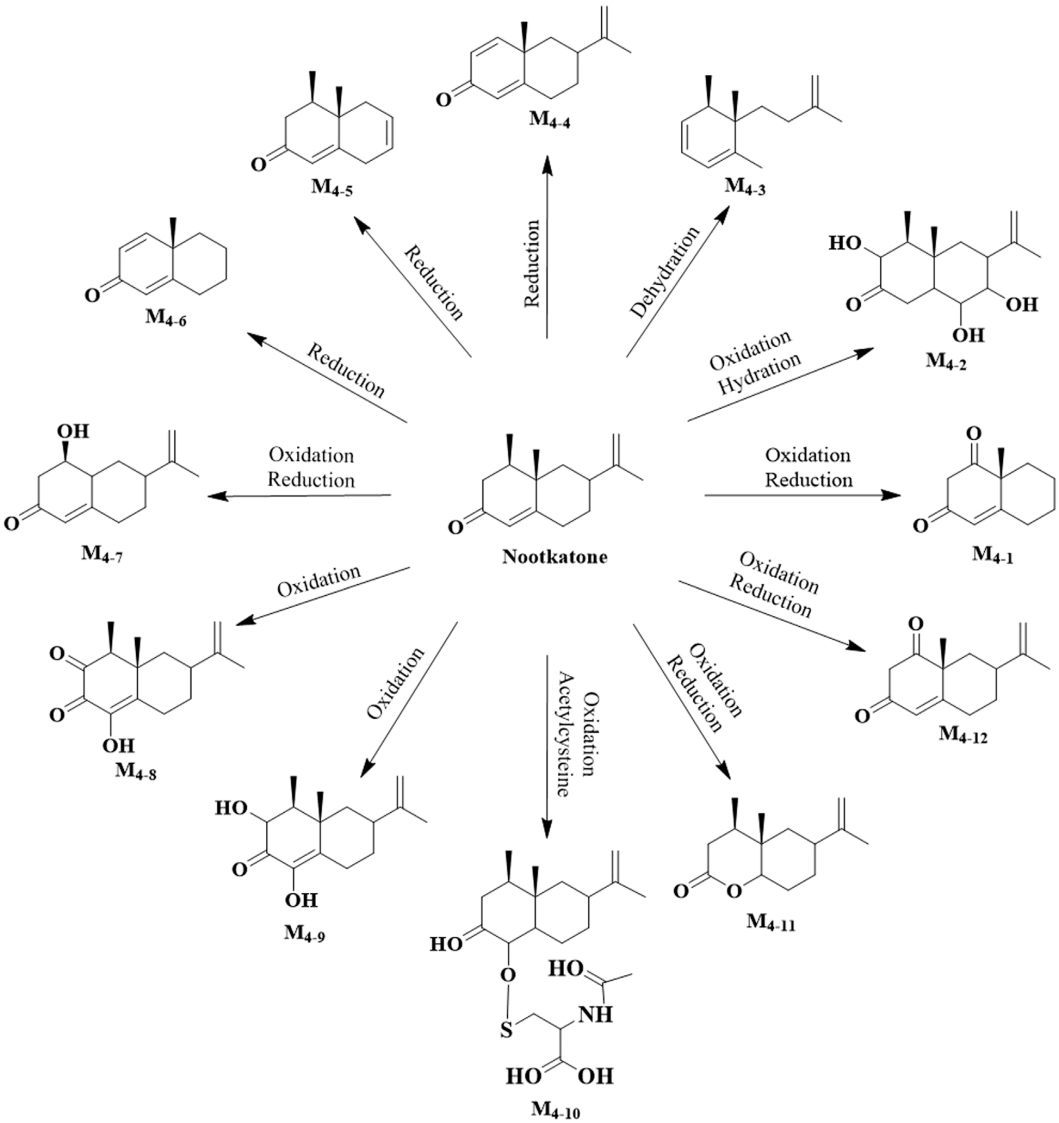
The proposed metabolic pathways of Nootkatone (M4).

In this study, 22 metabolites were identified across seven biological samples using Compound Discoverer software and referencing published literature (Zuo et al., 2021; Zhao, 2022). The tissue-specific distribution patterns of these metabolites are comprehensively illustrated in Figure 6. These metabolites were derived from the parent compounds Teuhetenone A (M_1_), Oxyphyllenodiol B (M_2_), Teuhetenone B (M_3_), and Nootkatone (M_4_). As schematically summarized, the proposed metabolic pathways of Nootkatone are delineated in Figure 7, whereas the proposed biotransformation pathways of the remaining three compounds are comprehensively mapped in Supplementary Figure S3. The major biotransformation pathways involved Phase I reactions, including oxidation, desaturation, dehydration, and reduction, with minor contributions from Phase II conjugation reactions such as glycine conjugation and acetylcysteine conjugation. Detailed information is provided in Supplementary Table S3.

The MB-W group exhibited the highest number of detected metabolites (14 metabolites), indicating that the intestine serves as the primary metabolic site for these four parent compounds, likely due to the abundance of metabolic enzymes in the intestinal wall. All nine metabolites identified in the MB-F group were also present in the MB-W group, suggesting their formation may involve enzymatic conversion mediated by the colonic wall; however, the potential contribution of gut microbiota cannot be excluded, and the precise mechanisms require further investigation. Among the 12 metabolites identified in the FVB group, only two were unique and absent in the MB-W group, implying these two metabolites are likely hepatic in origin, potentially mediated by hepatic drug-metabolizing enzymes. Eight metabolites were identified in the SGJ group, possibly generated via biotransformation of parent compounds under the acidic gastric environment and pepsin activity. 16 compounds were detected in the AA group, while a total of 8 metabolites were identified in the CSF and brain tissue groups. These findings demonstrate that most metabolites were able to enter systemic circulation, with a subset demonstrating BBB permeability or undergoing enzymatic metabolism in the CNS, highlighting their candidacy for further evaluation in CNS-targeted therapies. Further mechanistic and pharmacodynamic studies will be carried out to validate whether these metabolites indeed exert neuroprotective effects on the CNS.

### 3.3 SE-AOF ameliorates cognitive deficits, neuropathological changes, and biochemical abnor-malities in LPS-induced mice

#### 3.3.1 Behavioral tests

**FIGURE 8:**
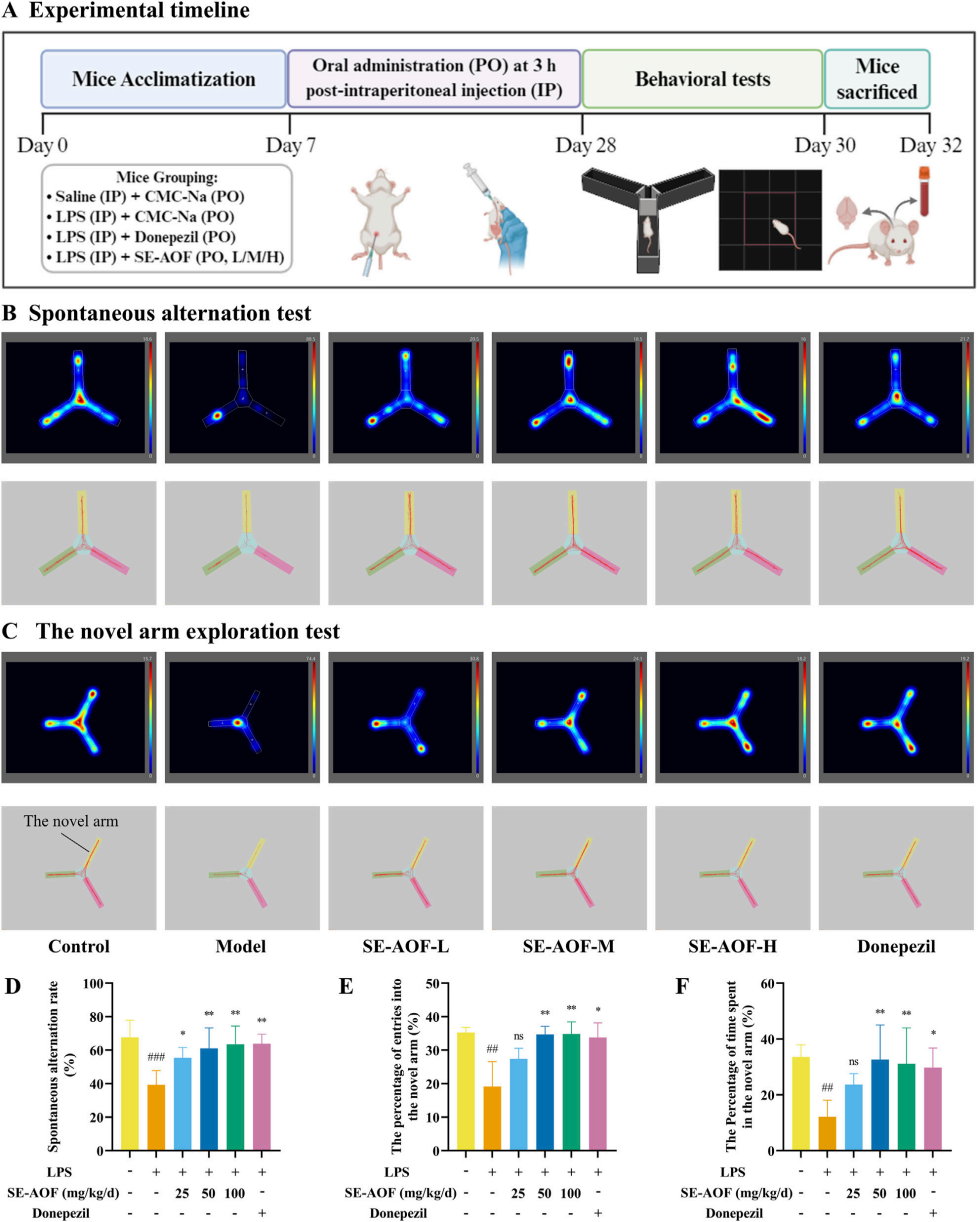
Experimental timeline and Y-maze test. **(A)** Schematic diagram of the experimental timeline for evaluating the protective effects of SE-AOF on LPS-induced cognitive impairment in mice. **(B)** Representative trajectories illustrating spontaneous alternation behavior in the different groups. **(C)** Example movement paths during novel arm exploration. **(D)** Percentage of spontaneous alternation. **(E)** Ratio of entries into the novel arm (%). **(F)** Percentage of total exploration time spent in the novel arm. ns indicates no significant difference. ##*p* < 0.01, ###*p* < 0.001, compared with the control group; *p < 0.05, **p < 0.01, compared with the model group.

Cognitive function was evaluated using the Y-maze spontaneous alternation and novel-arm exploration tests (Abdulbasit et al., 2018). Control mice exhibited high spontaneous alternation rates and a high frequency of entries into the novel arm, indicating intact spatial memory and exploratory behavior. By contrast, LPS-treated model mice showed a significant reduction in spontaneous alternation compared with controls (*p* < 0.001; Figures 8B,D). Treatment with low-, medium-, and high-dose SE-AOF and with donepezil significantly increased spontaneous alternation relative to the model group (*p* < 0.05, *p* < 0.01, *p* < 0.01, and *p* < 0.05, respectively). In the novel-arm exploration task, the percentage of entries into the novel arm was significantly lower in the model group than in controls (*p* < 0.01; Figures 8C,E). The medium- and high-dose SE-AOF groups and the positive control (donepezil) exhibited significantly higher percentages of novel-arm entries than the model group (*p* < 0.01, *p* < 0.01, and *p* < 0.05, respectively). Similarly, the proportion of time spent in the novel arm was reduced in the model group (*p* < 0.01; Figures 8C,F) and was significantly restored by medium- and high-dose SE-AOF and by donepezil (*p* < 0.01, *p* < 0.01, and *p* < 0.05, respectively).

**FIGURE 9:**
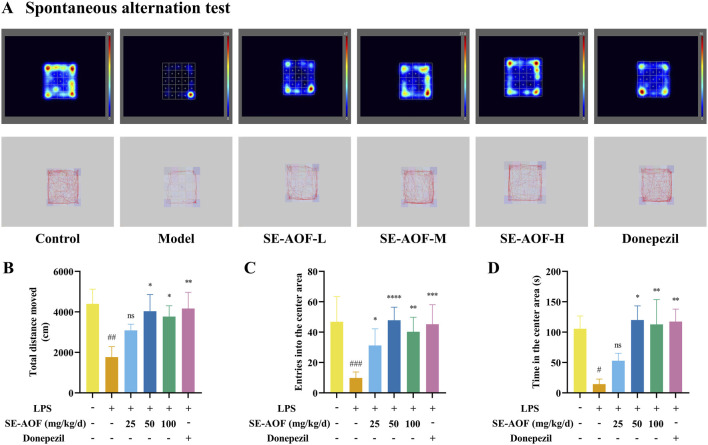
Open field test. **(A)** Representative movement trajectories of mice in each group. **(B)** Total distance traveled (cm). **(C)** Number of entries into the central zone. **(D)** Total time spent in the central zone (s). ns denotes no significant difference. #*p* < 0.05, ##*p* < 0.01, ###*p* < 0.001, compared with the control group; *p < 0.05, **p < 0.01, ***p < 0.001, ****p < 0.0001, compared with the model group.

Exploratory activity, locomotion, and anxiety-like behavior were assessed using the Open Field Test (Wan et al., 2024). Control mice displayed greater total distance traveled, more frequent center-zone entries, and longer time spent in the center, indicative of normal exploratory drive and affective state. Compared with controls, model mice exhibited significant decreases in total distance traveled, center-entry frequency, and time spent in the center (*p* < 0.01, *p* < 0.001, and *p* < 0.05, respectively; Figure 9). Medium- and high-dose SE-AOF, as well as donepezil, significantly ameliorated these LPS-induced behavioral deficits.

Taken together, administration of SE-AOF and donepezil attenuated LPS-induced cognitive impairment and behavioral abnormalities, improving learning and memory, affective state, and locomotor function in mice under the conditions of this study.

#### 3.3.2 H&E staining

**FIGURE 10:**
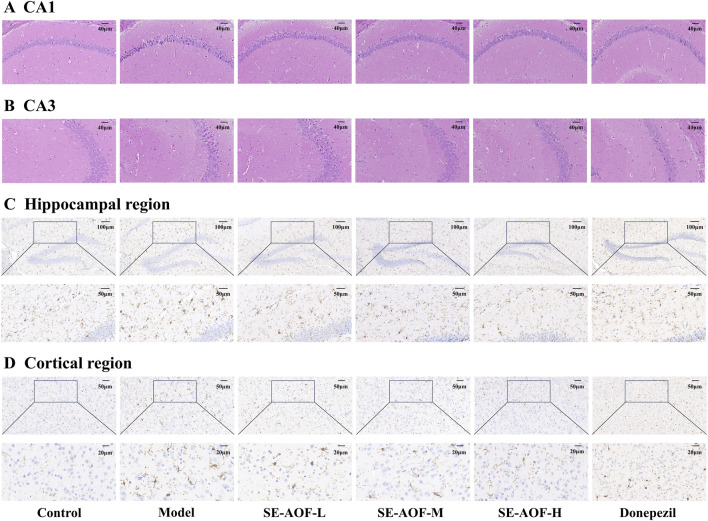
Histopathological and immunohistochemical evaluation of the brain. **(A)** Neural cell morphology in the hippocampal CA1 and **(B)** CA3 region (scale bar: 40 μm). **(C)** IBA-1 immunohistochemical staining in the hippocampus (scale bars: 100 μm, 50 μm) and **(D)** cortex (scale bars: 50 μm, 20 μm).

H&E staining was performed to evaluate histopathological alterations in hippocampal neurons of LPS-induced mice. In the control group, neurons in the CA1 and CA3 regions of the hippocampus were well-organized, exhibited normal morphology, and showed clear, round nuclei without evidence of pyknosis or cellular damage (Figures 10A,B). In contrast, the model group displayed pronounced pathological changes, characterized by nuclear pyknosis, disordered arrangement, shrunken cell bodies, and widened intercellular spaces, indicating structural damage to hippocampal neurons. Notably, treatment with SE-AOF or donepezil alleviated these abnormalities, as reflected by more orderly neuronal arrangement, improved cellular morphology, and reduced nuclear pyknosis, with the overall architecture approaching normal conditions. These findings suggest that both SE-AOF and donepezil exert protective effects against LPS-induced hippocampal injury.

#### 3.3.3 Inflammatory response in brain tissue

Neuroinflammation is recognized as a critical driver of disease progression, and microglia, the resident innate immune cells of the CNS, play a central role in this process (Perry et al., 2010). We assessed microglial abundance and morphology in the hippocampus and cortex using IBA-1 immunohistochemistry. In the control group, microglia predominantly exhibited a resting, ramified morphology characterized by small somata and thin, highly branched processes (Figures 10C,D). By contrast, the model group displayed a pronounced activated phenotype, with enlarged cell bodies, retracted and thickened processes, reduced branching, and a more rounded, amoeboid appearance, indicative of robust activation. Compared with the model group, SE-AOF treatments markedly attenuated microglial activation in both regions, as reflected by reduced soma hypertrophy, partial restoration of process length and branching, and a lower microglial density; donepezil produced similar but less pronounced changes. These findings indicate that SE-AOF significantly suppresses excessive microglial activation, thereby mitigating central neuroinflammatory responses.

#### 3.3.4 Inflammatory marker analysis

**FIGURE 11:**
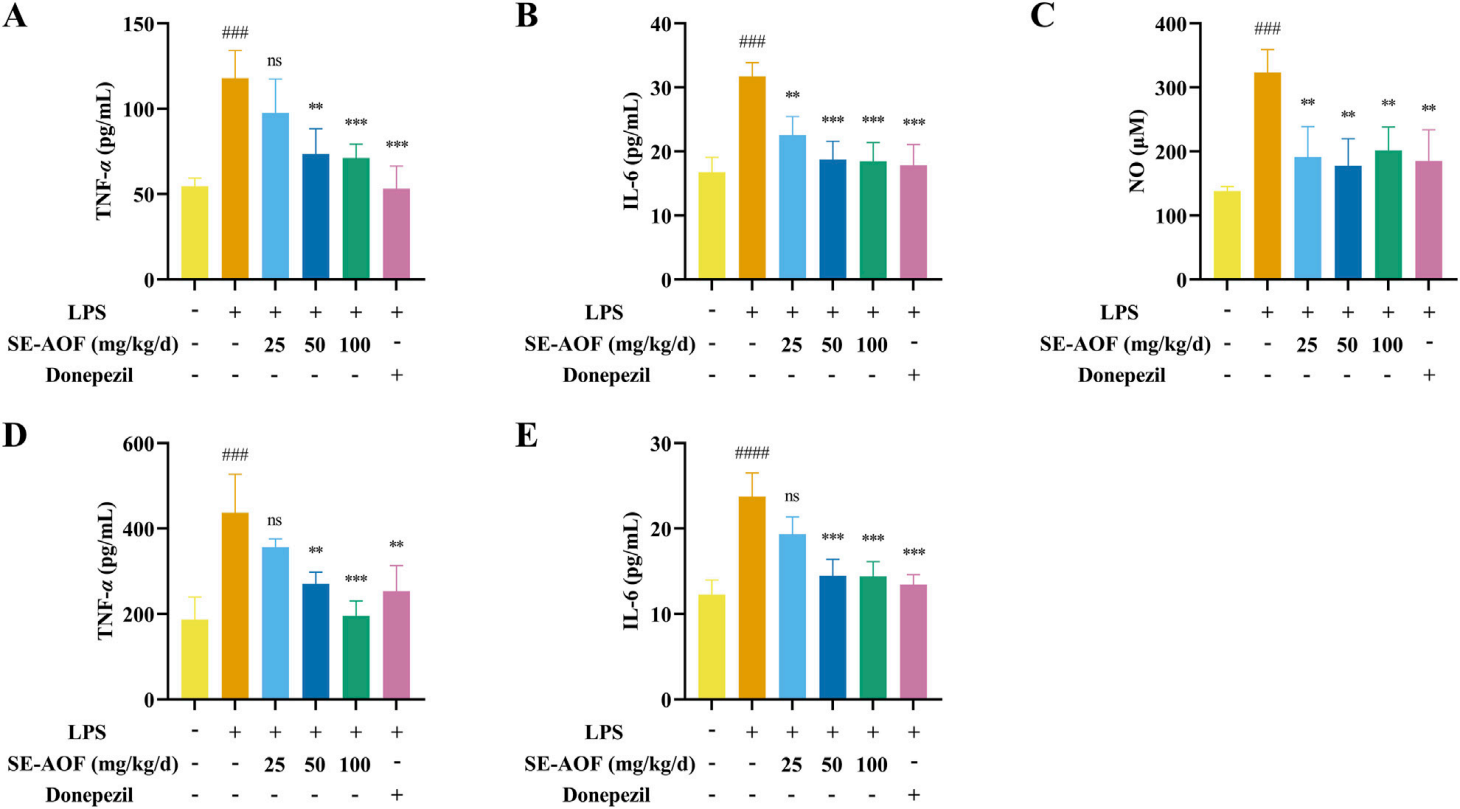
Levels of TNF-α the in plasma **(A)**, IL-6 in the plasma **(B)**, NO in the brain tissue **(C)**, TNF-α in the brain tissue **(D)**, and IL-6 in the brain tissue **(E)** in the different treatment groups. ns represents no significant difference. ###*p <* 0.001, ####*p <* 0.0001, compared with the control group. ns indicates no significant difference. **p < 0.01, ***p < 0.001, compared with the LPS group.

Plasma and brain tissue levels of representative inflammatory mediators were measured to assess systemic and central neuroinflammatory responses. Plasma levels of TNF-α and IL-6, together with tissue levels of TNF-α, IL-6 and NO in the brain, were measured using commercial assay kits according to the manufacturers’ protocols. As shown in Figure 11, LPS administration induced marked increases in plasma TNF-α and IL-6 compared with controls (both *p <* 0.001). Relative to the model group, medium- and high-dose SE-AOF and donepezil significantly reduced plasma TNF-α (*p* < 0.01, *p* < 0.001 and *p* < 0.001, respectively), while low-, medium- and high-dose SE-AOF as well as donepezil significantly decreased plasma IL-6 (*p <* 0.01, *p* < 0.001, *p* < 0.001 and *p* < 0.001, respectively). In brain tissue, NO, TNF-α and IL-6 were significantly elevated in model mice versus controls (*p* < 0.001, *p* < 0.001 and *p* < 0.0001, respectively). Treatment with low-, medium- and high-dose SE-AOF and donepezil significantly lowered brain NO levels (all *p* < 0.01). Brain TNF-α was significantly reduced by medium- and high-dose SE-AOF and by donepezil (*p* < 0.01, *p* < 0.001 and *p* < 0.01, respectively), and brain IL-6 was markedly decreased in the medium- and high-dose SE-AOF groups and in the donepezil group (all *p* < 0.001). Together, these data indicate that SE-AOF and donepezil attenuate both systemic and central inflammatory responses induced by LPS.

#### 3.3.5 Oxidative stress marker analysis

Inflammation is closely associated with oxidative stress, as excessive inflammatory responses could increase the generation of reactive oxygen species (ROS), leading to cellular damage and exacerbating neuronal injury (Fischer and Maier, 2015). In LPS-induced models, elevated levels of inflammatory mediators are often accompanied by enhanced oxidative stress; therefore, the activities of antioxidant enzymes such as SOD and CAT, along with levels of lipid peroxidation products such as MDA in plasma and brain tissue, provide a direct measure of systemic and central oxidative stress and are valuable for evaluating neuroprotective effects.

**FIGURE 12:**
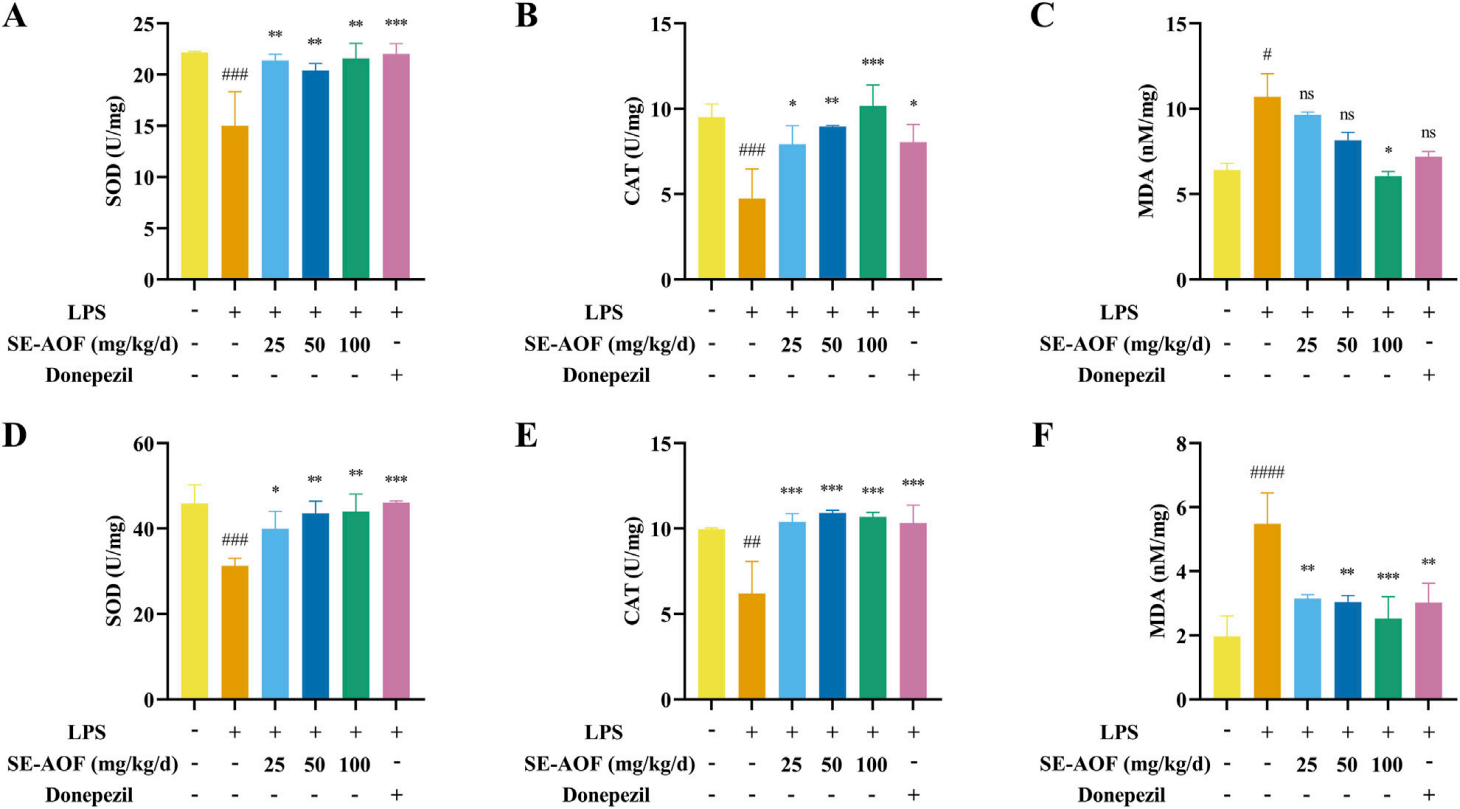
Levels of SOD in the plasma **(A)**, CAT in the plasma **(B)**, MDA in the plasma **(C)**, SOD in the brain tissue **(D)**, CAT in the brain tissue **(E)**,and MDA in the brain tissue **(F)** in the different treatment groups. ns represents no significant difference. ###*p* < 0.001, ####*p* < 0.0001, compared with the control group. ns indicates no significant difference. **p < 0.01, ***p < 0.001, compared with the LPS group.

Accordingly, in this study, the activities of SOD and CAT, as well as MDA levels, were measured in the plasma and brain. (Figure 12). Following LPS administration, the plasma activities of SOD and CAT in the model group were markedly decreased (both *p* < 0.001), whereas MDA levels were significantly increased (*p* < 0.05), indicating elevated systemic oxidative stress. Compared with the model group, low-, medium-, and high-dose SE-AOF treatments significantly increased the plasma activities of SOD (all *p* < 0.01) and CAT (*p* < 0.05, P < 0.01, *p* < 0.001), while only the high-dose SE-AOF group exhibited a significant reduction in the MDA level (*p* < 0.05). In the donepezil group, the plasma activities of SOD and CAT were significantly elevated (*p* < 0.001 and *p* < 0.05), whereas MDA levels showed no significant change.

In brain tissue, the activities of SOD and CAT were significantly decreased (*p* < 0.001 and *p* < 0.01), and MDA levels were markedly increased (*p* < 0.0001) in the model group, reflecting pronounced central oxidative stress. Compared with the model group, low-, medium-, and high-dose SE-AOF as well as donepezil treatments significantly increased brain SOD activity (*p* < 0.05, *p* < 0.01, *p* < 0.01, *p* < 0.001) and CAT activity (all *p* < 0.001); moreover, MDA levels in brain tissue were significantly reduced by medium- and high-dose SE-AOF and donepezil (*p* < 0.01, *p* < 0.01, *p* < 0.001, *p* < 0.01).

Although the primary focus of this study is central neuroinflammation, previous evidence suggests that systemic inflammatory status could substantially affect neuroinflammatory responses (Sun et al., 2022; Walker et al., 2023). Measurement of inflammatory cytokines and oxidative stress markers in plasma may therefore provide useful indications of peripheral immune and oxidative status, helping to infer potential interactions between peripheral and central inflammation. Based on the present findings and existing literature, it is plausible that SE-AOF exerts modulatory effects on both systemic and central inflammatory responses, which may together contribute to the attenuation of LPS-induced neuronal injury. Further studies will be conducted to clarify how peripheral inflammation influences central neuroinflammatory processes and to elucidate the underlying mechanisms of SE-AOF in regulating neuroinflammation.

## 4 Conclusion

This study systematically characterized the chemical constituents and metabolic dynamics of AOF through an integrated UPLC-HRMS/FBMN approach combined with sequential metabolism analysis. A total of 108 constituents were annotated, with 63 prototypes and 22 metabolites identified *in vivo*. Notably, 34 compounds, predominantly sesquiterpenoids, were detected in CSF and brain tissue, confirming their BBB permeability and supporting the traditional CNS-related applications of AOF. Importantly, pharmacological evaluation demonstrated that sesquiterpenoids of AOF significantly alleviated LPS-induced neuroinflammation-associated cognitive impairment in mice, as evidenced by improvements in behavioral performance, attenuation of microglial activation, and normalization of inflammatory and oxidative stress markers in both plasma and brain tissue. These findings not only provide pharmacological evidence for the therapeutic potential of AOF in neuroinflammatory and neurodegenerative disorders but also establish a transferable analytical framework for studying medicine–food homologous plants.

## Data Availability

The original contributions presented in the study are included in the article/Supplementary Material, further inquiries can be directed to the corresponding authors.

## Abbreviations

AOF, Alpiniae Oxyphyllae Fructus; BBB, blood-brain barrier; CAT, Catalase; CNS, central nervous system; FBMN, Feature-based molecular networking; GNPS, Global Natural Products Social Molecular Networking; H&E, Hematoxylin-eosin; IL-6, interleukin-6; IPVS, intestinal perfusion with venous sampling; MDA, malondialdehyde; NO, nitric oxide; SE-AOF, sesquiterpenoid-enriched fractions from Alpiniae Oxyphyllae Fructus; SEM, standard error of the mean; SOD, superoxide dismutase; TNF-α, tumor necrosis factor-α.
