# Supplementary material for: Identification, biotransformation, and neuroprotective potential of the ethanol extract of Alpiniae oxyphyllae fructus in neuroinflammation-related cognitive impairment
Source: Front Pharmacol. 2025 Nov 26;16:1714500. doi: 10.3389/fphar.2025.1714500 (PMC12689551; doi:10.3389/fphar.2025.1714500)
Supplement: Supplementary file 1 [file DataSheet1.zip › Supplementary Material/Supplementary Material.docx]

Supplementary Material

# Supplementary Figures and Tables

## Supplementary Figures

***
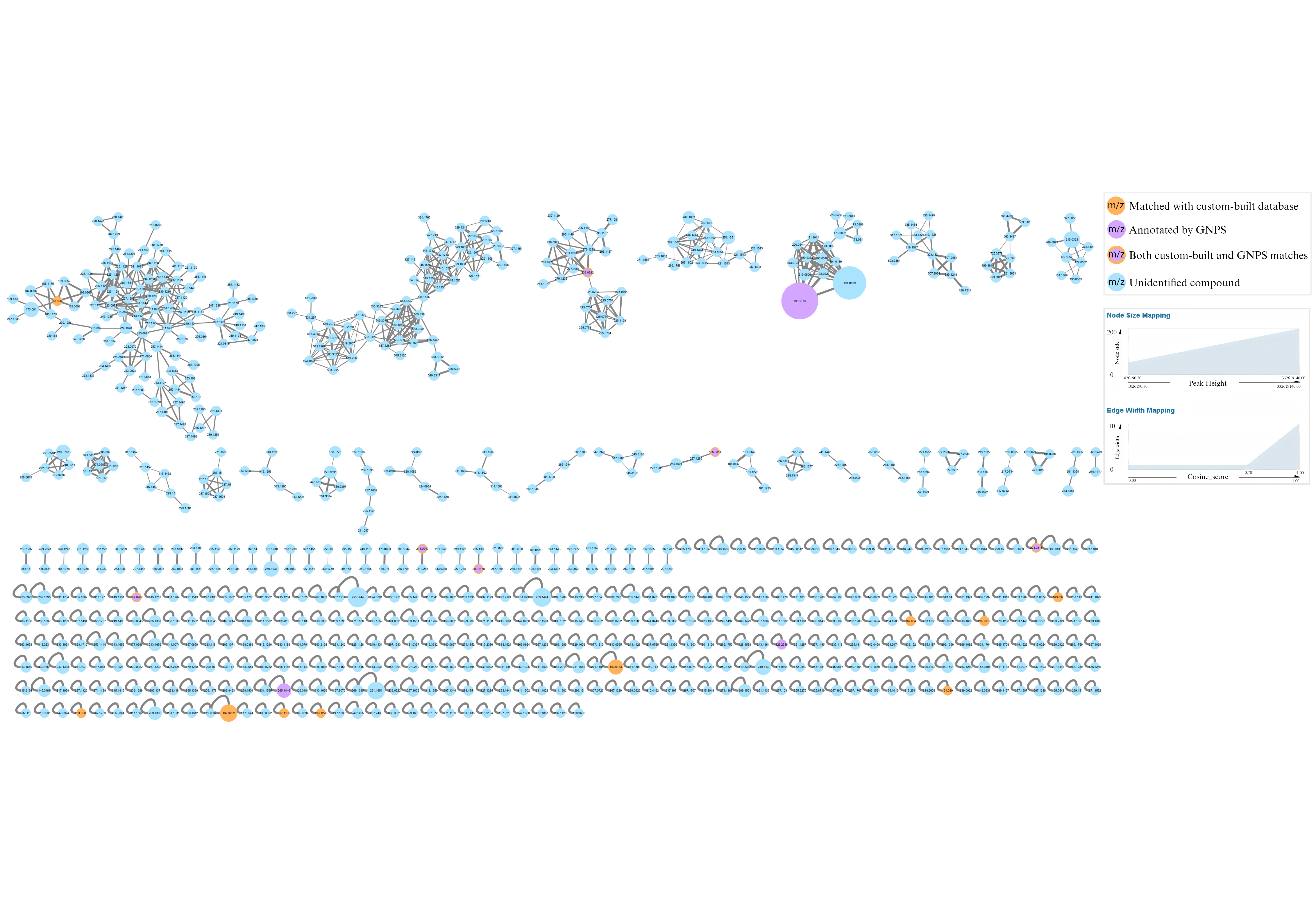
***

**Supplementary Figure 1.** The feature-based molecular networking diagram of AOF in negative ion mode.





**Supplementary Figure 2.** Base peak intensity (BPI) chromatograms of AOF extract from different biological samples: (A) simulated gastric juice; (B) mesenteric blood from intestinal wall metabolism group; (C) mesenteric blood from intestinal flora metabolism group; (D) femoral venous blood from hepatic metabolism; (E) abdominal aorta; (F) cerebrospinal fluid; (G) brain tissue; in each figure, black denotes the positive ion mode and brown represents the negative ion mode.


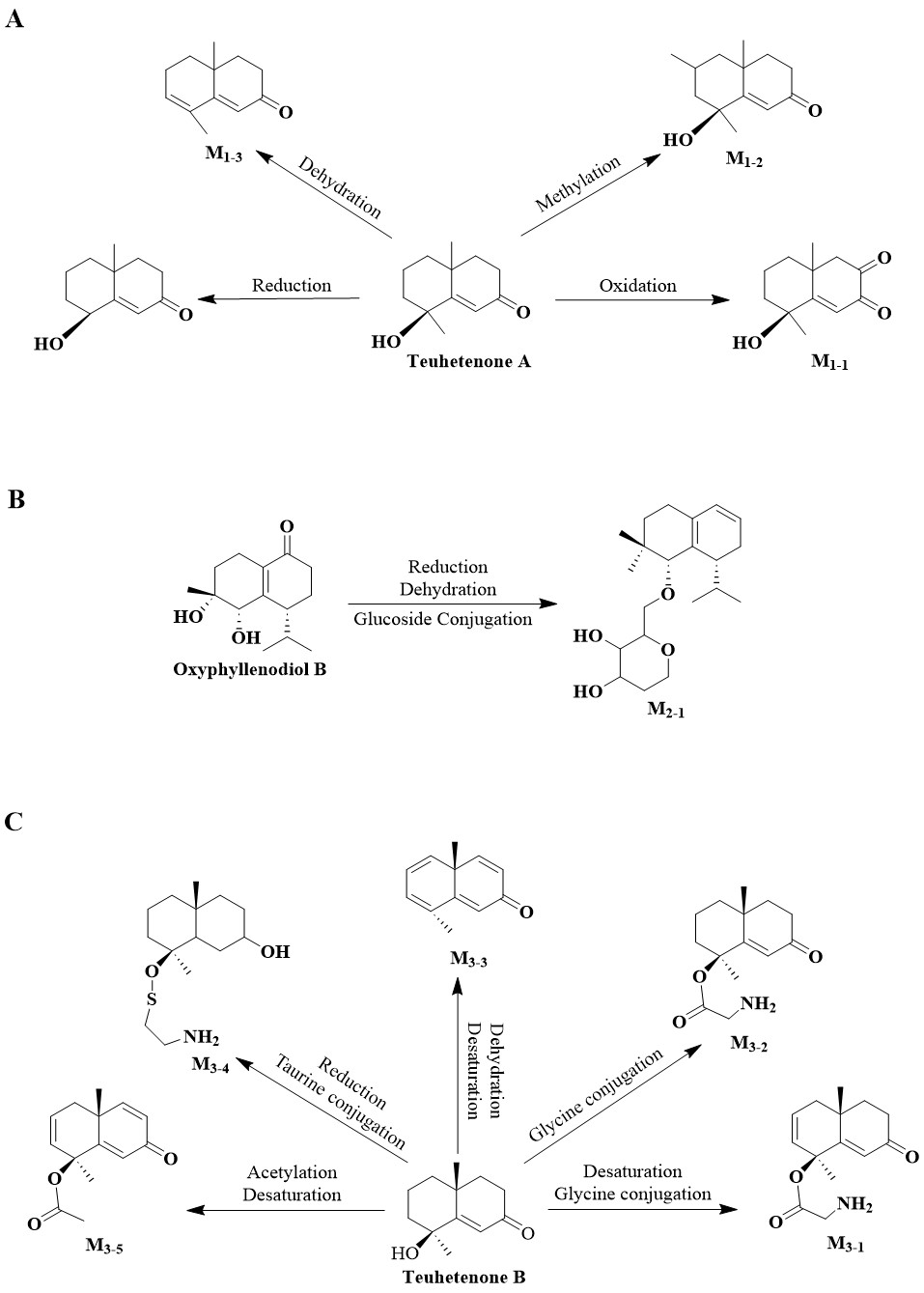


**Supplementary Figure 3.** The proposed metabolic pathways of Teuhetenone A(M1), Oxyphyllenodiol B (M2), Teuhetenone B (M3).

## Supplementary Tables

**Supplementary Table 1. Identification of components in AOF by UPLC-HRMS.**

| Peak  NO. | *t*_R_ (min) | Observed Mass | Error (ppm) | MS/MS Fragments | Molecular  Formula | Proposed Compound | Type | Reference/  database | |
| --- | --- | --- | --- | --- | --- | --- | --- | --- | --- |
| 1^a^ | 0.81 | [M+H]^+^ 266.1234 | 0.044 | 248.1131, 116.0710, 98.0606, 87.0447 | C_10_H_19_NO_7_ | D-1-[(3-Carboxypropyl) amino]-1-deoxyfructose | Organic  acids | - | |
| 2 | 0.84 | [M+H]^+^ 182.0813 | 0.551 | 136.0759, 123.0446, 119.0494, 91.0549 | C_9_H_11_NO_3_ | L-Tyrosine | Amino acid | Pubchem | |
| 3^a^ | 0.85 | [M-H]^-^  191.0189 | 1.628 | 191.0189, 111.0074, 87.0073, 85.0281 | C_6_H_8_O_7_ | Citric acid | Organic acids | - | |
| 4^b^ | 0.93 | [M+H]^+^ 204.0868 | -3.761 | 204.0868, 156.0659, 97.0290 | C_9_H_17_NS_2_ | 1-Isothiocyanato-7-(me-thylthio) heptane | Others | Pubchem | |
| 5 | 0.96 | [M+H]^+^ 123.0556 | 2.118 | 123.0556, 106.0292, 96.0450, 80.0502, 78.0345 | C_6_H_6_N_2_O | Nicotinamide | Others | (Inamadugu et al., 2010) | |
| 6 | 0.99 | [M+H]^+^ 124.0396 | 1.975 | 125.0430, 124.0396, 80.0502 | C_6_H_5_NO_2_ | Nicotinic acid | Organic acids | (Lang et al., 2008) | |
| 7^a^ | 0.99 | [M+NH_4_-H_2_O]^+^ 180.0867 | 0.116 | 134.0967, 127.0393, 97.0290, 85.0291, | C_6_H_12_O_6_ | D-Fructose | Others | - |  |
| 8 | 1.05 | [M+H]^+^ 268.1039 | -0.337 | 136.0620, 119.0358, 85.0291, 57.0344, 55.0187 | C_10_H_13_N_5_O_4_ | Adenosine | Others | Pubchem |  |
| 9 | 1.15 | [M+H]^+^ 284.0988 | -0.686 | 153.0615, 152.0569, 135.0302,  110.0354, 109.0509 | C_10_H_13_N_5_O_5_ | Guanosine | Others | Pubchem |  |
| 10 | 1.25 | [M+H]^+^ 190.1074 | 0.029 | 190.1231, 144.1021 | C_8_H_15_NO_4_ | 2-Aminooctanedioic acid | Amino acid | (von Bargen et al., 2013) |  |
| 11 | 1.33 | [M+H]^+^ 199.1077 | 0.106 | 170.0806, 112.0398, 83.0498, 55.0186 | C_9_H_14_N_2_O_3_ | Metharbital | Amino acid | Pubchem |  |
| 12^a^ | 1.36 | [M+H]^+^ 166.0863 | 0.451 | 166.0865, 149.0601, 121.0846, 120.0812, 103.0547 | C_9_H_11_NO_2_ | Phenylalanine | Others | Pubchem |  |
| 13 | 1.98 | [M+H]^+^ 260.0917 | -0.248 | 199.0623, 184.0963 | C_14_H_13_NO_4_ | Skimmianine | Alkaloid | (Jeong et al., 2021) |  |
| 14 | 2.26 | [M+H]^+^ 127.0392 | 1.412 | 127.0392, 109.0288, 81.0342 | C_6_H_6_O_3_ | 5-Hydroxymethylfurfural | Aldehydes | * |  |

Supplementary Table 1 (Continued)

| Peak  NO. | *t*_R_ (min) | Observed Mass | Error (ppm) | MS/MS Fragments | Molecular  Formula | Proposed Compound | Type | Reference/  database | |
| --- | --- | --- | --- | --- | --- | --- | --- | --- | --- |
| 15 | 2.31 | [M-H]^-^  167.034 | 0.687 | 167.0339, 152.0110, 123.0438 | C_8_H_8_O_4_ | Vanillic acid | Organic acids | (Qi et al., 2024) |  |
| 16 | 2.45 | [M+H]^+^ 143.034 | 0.803 | 143.0341, 97.0290, 69.0343 | C_6_H_6_O_4_ | Kojic acid | Organic acids | Pubchem |  |
| 17^a^ | 2.51 | [M+H]^+^ 205.0972 | 0.418 | 188.0708, 144.0810, 146.0603, 118.0655 | C_11_H_12_N_2_O_2_ | L-Tryptophan | Amino acid | - |  |
| 18^a^ | 2.51 | [M+H]^+^ 367.1499 | -0.129 | 229.0974, 188.0707, 146.0602, 144.0809 | C_17_H_22_N_2_O_7_ | tryptophan N-glucoside | Others | - |  |
| 19 | 2.88 | [M+Na]^+^ 1283.1751 | -0.281 | 177.0912, 133.0649, 89.0606 | C_12_H_26_O_7_ | Hexaethyleneglycol | Others | Pubchem |  |
| 20^b^ | 3.15 | [M-H]^-^  211.0243 | 2.965 | 167.0339, 137.0230, 123.0438, 93.0331, 65.0382 | C_9_H_8_O_6_ | 5-Carboxyvanillic acid | Organic acids | (Qi et al., 2024) |  |
| 21 | 3.27 | [M-H]^-^  153.0183 | 0.162 | 153.0182, 109.0281 | C_7_H_6_O_4_ | Protocatechuic acid | Organic acids | * |  |
| 22 | 3.46 | [M-H]^-^  138.0185 | -0.06 | 138.0550, 94.0284, 93.0331 | C_6_H_5_NO_3_ | 6-Hydroxynicotinic acid | Organic acids | (Qi et al., 2024) |  |
| 23 | 3.80 | [M-H]^-^  137.0232 | -0.953 | 137.0232, 136.0154, 119.0118, 109.0282, 108.0202 | C_7_H_6_O_3_ | 3,4-Dihydroxybenzaldehyde | Aldehydes | pubchem |  |
| 24^a^ | 4.03 | [M-H_2_O+H]^+^ 151.1119 | 0.717 | 151.1120, 133.1014, 107.0860, 93.0705 | C_10_H_16_O_2_ | Jasmine lactone | Esters | - |  |
| 25 | 4.03 | [M+H]^+^ 211.1329 | 0.280 | 193.1225, 175.119, 157.1018, 151.1120, 133.1014 | C_12_H_18_O_3_ | Oxyphyllenone A | Terpenes | (Zhang, 2020) |  |
| 26^a^ | 4.06 | [M+H]^+^ 231.1128 | 0.025 | 188.0707, 168.0810, 158.0967, 130.0654 | C_13_H_14_N_2_O_2_ | Tetrahydroharman-3-carboxylic acid | Organic acids | - |  |
| 27^a^ | 4.12 | [M+H]^+^ 291.0974 | -0.577 | 134.0449, 130.0654, 88.0400, 74.0245 | C_14_H_14_N_2_O_5_ | Indoleacetylaspartate | Others | - |  |
| 28 | 4.20 | [M-H]^-^  271.0823 | 4.097 | 113.0231, 101.0590, 89.0230, 71.0124, 59.0124 | C_12_H_16_O_7_ | Arbutin | Others | (Qi et al., 2024) |  |
| 29 | 4.39 | [M-H]^-^  151.039 | 0.128 | 151.0389, 136.0153, 123.0437 | C_8_H_8_O_3_ | Isovanillin or isomer | Aldehydes | pubchem |  |

Supplementary Table 1 (Continued)

| Peak  NO. | *t*_R_ (min) | Observed Mass | Error (ppm) | MS/MS Fragments | Molecular  Formula | Proposed Compound | Type | Reference/  database |
| --- | --- | --- | --- | --- | --- | --- | --- | --- |
| 30 | 4.62 | [M+H]^+^ 193.1224 | 0.382 | 175.1119, 165.1274,147.1170, 123.0807, 105.0704 | C_12_H_16_O_2_ | Oxyphyllanene A | Terpenes | (Zhang, 2020) |
| 31^b^ | 4.87 | [M+H]^+^ 191.1066 | -0.399 | 191.1067, 173.0963,163.1119, 145.1013, 135.0806, 119.0860 | C_12_H_14_O_2_ | Oxyphyllanene B | Terpenes | (Zhang, 2020) |
| 32^b^ | 4.98 | [M-H]^-^  239.0922 | 3.304 | 221.0820, 196.1055, 195.1020, 151.1117 | C_12_H_16_O_5_ | 3-carboxy-4-methyl-5-propyl-2-Furanpropionic acid | Organic acids | Pubchem |
| 33^ab^ | 5.19 | [M-H]^-^  187.0967 | 1.307 | 187.0967, 169.0862, 125.0959, 123.0801, 97.0643 | C_9_H_16_O_4_ | Azelaic acid | Organic acids | Pubchem massbank |
| 34 | 5.25 | [M-H]^-^  577.1359 | 3.132 | 289.0719, 425.0883, 407.0773, 125.0231 | C_30_H_26_O_12_ | Procyanidin B1 | Flavonoids | (Ruan, 2021) |
| 35^a^ | 5.39 | [M-H]^-^  289.0719 | 4.170 | 245.0817, 203.0707, 151.0390, 109.0282 | C_15_H_14_O_6_ | (+)-Catechin | Flavonoids | (Kong et al., 2024) |
| 36^b^ | 5.42 | [M+H]^+^ 167.1066 | -0.097 | 149.0963, 126.0551, 93.0705 | C_10_H_14_O_2_ | Perillic acid | Organic acids | (Kong et al., 2024) |
| 37^b^ | 5.46 | [M+H]^+^ 211.1329 | 0.090 | 193.1225, 175.119, 151.1120, 133.1014 | C_12_H_18_O_3_ | Oxyphyllenone B | Terpenes | (Sun et al., 2016) |
| 38 | 5.66 | [M-H]^-^  151.039 | 0.195 | 151.0389, 136.0153, 123.0438 | C_8_H_8_O_3_ | Isovanillin or isomer | Aldehydes | pubchem |
| 39 | 6.01 | [M+H]^+^ 237.1484 | -0.553 | 219.1382, 201.1273, 191.1431, 173.1326, 131.0857, 105.0703 | C_14_H20O3 | Oxyphyllone E | Terpenes | (Zhang, 2020) |
| 40^b^ | 6.11 | [M+H]^+^ 267.1589 | -0.658 | 267.1590, 249.1486, 231.1381, 213.1275 | C15H22O4 | Verrucarol | Terpenes | Massbank |
| 41 | 6.20 | [M+H]^+^ 239.1641 | -0.381 | 221.1538, 203.1433, 193.1590, 175.1483, 161.1325, 147.0808, | C14H22O3 | Oxyphyllenodiol A | Terpenes | (Zhang, 2020) |
| 42^b^ | 6.21 | [M-H]^-^  185.081 | 0.997 | 185.081, 141.0909 | C_9_H_14_O_4_ | 1-Carboxycyclohexaneacetic Acid | Organic acids | Pubchem |
| 43 | 6.23 | [M+H]^+^ 165.0911 | 0.750 | 147.0806, 119.0859, 105.0704 | C10H12O2 | Eugenol | Organic acids | (Qi et al., 2024) |
| 44 | 6.25 | [M-H]^-^  179.0341 | 0.976 | 135.0437, 134.0595, 117.0178 | C_9_H_8_O_4_ | Caffeate | Organic acids | (Patras et al., 2018) |
| 45^b^ | 6.55 | [M-H]^-^  221.0816 | 3.278 | 221.0816,177.0912,121.0278,71.0121 | C_12_H_14_O_4_ | Monobutyl phthalate or isomer | Esters | Pubchem\massbank |
| 46^b^ | 6.69 | [M+H]^+^ 253.1797 | -0.360 | 235.1695, 217.1589, 205.1588, 189.1275, 177.1276 | C15H24O3 | (11S)-Nootkatone-11,12-diol | Terpenes | (Zhang, 2020) |

Supplementary Table 1 (Continued)

| Peak  NO. | *t*_R_ (min) | Observed Mass | Error (ppm) | MS/MS Fragments | Molecular  Formula | Proposed Compound | Type | Reference/  database |
| --- | --- | --- | --- | --- | --- | --- | --- | --- |
| 47 | 6.76 | [M+H]^+^ 283.1539 | -0.460 | 265.1435, 219.1380, 205.1225, 201.1274, 187.1116, 161.0961 | C15H22O5 | Artemisinin or isomer | Terpenes | (Qi et al., 2024) |
| 48 | 6.79 | [M+H]^+^ 253.1797 | -0.479 | 235.1695, 217.1589, 205.1588, 189.1275, 177.1276 | C15H22O2 | (11R)-Nootkatone-11,  12-diol | Terpenes | (Zhang, 2020) |
| 49 | 7.07 | [M+H]^+^ 225.1122 | 0.064 | 207.1019, 189.0912, 161.0963, 133.1014, 91.0550 | C12H16O4 | Senkyunolide H | Esters | (Wu et al., 2024) |
| 50 | 7.24 | [M-H]^-^  163.039 | 0.180 | 119.0489, 117.0338, 91.0539 | C9H8O3 | *cis*-4-Coumaric acid | Organic acids | (Ruan, 2021) |
| 51 | 7.59 | [M+H]^+^ 135.1169 | 0.318 | 135.1170, 119.0862, 107.0861, 105.0705, 93.0705 | C_10_H_14_ | p-Cymene | Terpenes | (Sun et al., 2016) |
| 52^b^ | 7.62 | [M+H]^+^ 195.1380 | 0.224 | 177.1276, 159.1171, 149.1327, 137.0963, 121.0651, 107.0861, | C12H18O2 | Teuhetenone A | Terpenes | (Patras et al., 2018) |
| 53^ab^ | 7.82 | [M-H]^-^  187.0967 | 1.361 | 187.0967,169.0859,125.0959,123.0803,97.0645 | C9H16O4 | Azelaic acid | Organic acids | Pubchem |
| 54 | 7.84 | [M+H]^+^ 261.1120 | -0.404 | 243.1010, 215.1068, 197.0964, 187.1118, 169.1013, | C15H16O4 | 8-Deoxylactucin | Terpenes | Pubchem |
| 55^b^ | 7.87 | [M+H]^+^ 239.1641 | -0.339 | 221.1538, 203.1433, 193.1590, 179.1432 | C14H22O3 | Oxyphyllenodiol B | Terpenes | (Zhang, 2020) |
| 56 | 7.96 | [M+H]^+^ 237.1485 | -0.089 | 219.1383, 201.1275, 191.1432, 173.1326, 159.1170, 131.0858 | C14H20O3 | (5S,7R,10R)-5-hydroxy-noreudesma-2-tien-3,11-dione | Terpenes | (Zhang, 2020) |
| 57^ab^ | 8.29 | [M-H]^-^  209.1178 | 2.674 | 209.1178, 165.1274, 59.0124 | C_12_H_18_O_3_ | Jasmonic acid | Organic acids | (Enomoto and Miyamoto, 2021) |
| 58^b^ | 8.55 | [M+H]^+^ 195.1381 | 0.685 | 177.1276, 159.1169, 149.1326, 137.0963, 121.0651, 107.0860 | C12H18O2 | Teuhetenone B | Terpenes | (Zhang, 2020) |
| 59 | 8.77 | [M+H]^+^ 239.2005 | -0.446 | 239.2009, 203.1796, 175.1482, 161.1327, 119.0859, 105.0703 | C15H26O2 | Oplopanone | Terpenes | (Zhang, 2020) |
| 60^b^ | 8.86 | [M+H]^+^ 219.1380 | 0.290 | 191.1429, 173.1327, 131.0858, 105.0704 | C14H18O2 | Oxyphyllone D | Terpenes | (Zhang, 2020) |
| 61^b^ | 8.89 | [M+H]^+^ 235.1693 | -0.028 | 235.1694, 217.1588,199.1484,  189.1639, 149.0963, 121.1015 | C15H22O2 | 7-epi-Teucrenone | Terpenes | (Zhang, 2020) |
| 62^b^ | 8.95 | [M+H]^+^ 283.1538 | -0.672 | 265.1434, 219.1381, 205.1226, 201.1273, 187.1120, 161.0963 | C15H22O5 | Artemisinin or isomer | Terpenes | (Qi et al., 2024) |

Supplementary Table 1 (Continued)

| Peak  NO. | *t*_R_ (min) | Observed Mass | Error (ppm) | MS/MS Fragments | Molecular  Formula | Proposed Compound | Type | Reference/  database |
| --- | --- | --- | --- | --- | --- | --- | --- | --- |
| 63 | 8.98 | [M+H]^+^ 265.1433 | -0.361 | 168.0934, 155.0856, 153.0913, 142.0784, 129.0705 | C15H20O4 | (3aR,4R,5aS,6S,9aR,9bS)-4,6-dihydroxy-5a,9-dimethyl-3-methylidene-4,5,6,7,9a,9b-hexahydro-3aHbenzo[g][1] Benzofuran-2-one | Others | Pubchem |
| 64^b^ | 9.48 | [M+NH_4_]^+^ 270.2063 | -0.260 | 235.1693, 217.1589, 199.1483, 189.1638, 133.1014 | C15H24O3 | 2-(8-Hydroxy-4a,8-dimethyldecahydro-2-naph-thalenyl) acrylic acid | Organic acids | Pubchem |
| 65^b^ | 9.57 | [M+H]^+^ 221.1537 | 0.242 | 203.1433, 161.0963 | C14H20O2 | 2,6-Di-tert-butyl-1,4-benzoquinone | Others | Pubchem |
| 66 | 9.64 | [M+H]^+^ 135.0806 | 0.951 | 135.0807, 107.0860, 91.0548 | C9H10O | Chavicol | Organic acids | (Qi et al., 2024) |
| 67^ab^ | 9.73 | [M+H]^+^ 233.1536 | 0.015 | 233.1538, 215.1434, 131.0858 | C15H20O2 | Costunolide | Terpenes | (Qi et al., 2024) |
| 68^b^ | 10.10 | [M+H]^+^ 237.1849 | -0.112 | 219.1719, 201.1640, 135.1172 | C15H24O2 | Capsidiol | Terpenes | (Qi et al., 2024) |
| 69^ab^ | 10.44 | [M+H]^+^ 235.1693 | -0.028 | 235.1694, 217.1588, 177.1275, 175.1118, 119.0858 | C15H22O2 | Curcumenol | Terpenes | (Qi et al., 2024) |
| 70 | 10.70 | [M-H]^-^  193.1226 | 1.521 | 193.1225, 151.0752, 108.0444 | C_12_H_18_O_2_ | 4-Hexylresorcinol | Organic acids | Pubchem |
| 71^b^ | 11.01 | [M-H]^-^  221.0816 | 3.323 | 221.0816, 177.0912, 121.0643, 71.0483 | C_12_H_14_O_4_ | Monobutyl phthalate or isomer | Esters | Puchem/massbank |
| 72^b^ | 11.12 | [M+H]^+^ 217.1587 | 0.130 | 199.1484, 189.1639, 175.1119, 161.0962, 147.0803, 133.1014 | C15H20O | Dehydronootkatone | Terpenes | (Zhang, 2020) |
| 73 | 11.13 | [M-H]^-^  193.1226 | 1.676 | 193.1225, 151.0752, 108.0439 | C_12_H_18_O_2_ | 4-Hexylresorcinol isomer | Organic acids | Pubchem |
| 74 | 11.65 | [M+H]^+^ 265.1434 | -0.021 | 155.0862, 153.0908, 142.0782, 129.0699 | C15H20O4 | (3aR,4R,5aS,6S,9aR,9bS)-4,6-dihydroxy-5a,9-dimethyl-3-methylidene-4,5,6,7,9a,9b-hexahydro-3aH-benzo[g][1] benzo-furan-2-one isomer | Others | Pubchem |

Supplementary Table 1 (Continued)

| Peak  NO. | *t*_R_ (min) | Observed Mass | Error (ppm) | MS/MS Fragments | Molecular  Formula | Proposed Compound | Type | Reference/  database |
| --- | --- | --- | --- | --- | --- | --- | --- | --- |
| 75 | 12.03 | [M-H]^-^  207.1021 | 2.410 | 207.1064, 189.0917, 163.1113 | C_12_H_16_O_3_ | *β*-asarone | Others | (Zhang, 2020) |
| 76 | 12.26 | [M+H]^+^ 223.1692 | -0.164 | 223.1695, 205.1589, 187.1480, 163.1118, 151.1118, 145.1013 | C14H22O2 | Oxyphyllenone H or isomer | Terpenes | (Zhang, 2020) |
| 77 | 12.33 | [M+H]^+^ 249.1484 | -0.406 | 231.1382, 185.1324, 161.0962 | C15H20O3 | Parthenolide | Terpenes | (Qi et al., 2024) |
| 78^a^ | 12.37 | [M-H]^-^  329.2334 | 3.582 | 329.2335, 211.1336, 171.1017, 139.1115 | C_18_H_34_O_5_ | 9,12,13-Trihydroxyoctadec-10-enoic acid | Organic acids | - |
| 79 | 12.54 | [M+H]^+^ 235.1692 | -0.070 | 177.0927, 147.0806, 119.0859 | C15H22O2 | (+) - (4R,5S,7R)-13-Hy-droxynootkatone | Terpenes | (Zhang, 2020) |
| 80 | 12.84 | [M-H]^-^  307.1189 | 4.185 | 149.0596, 147.0438, 105.0329, 123.0801, 107.0484 | C_16_H_20_O_6_ | 5,13,15-Trihydroxy-9-methyl-10-oxabicyclo [10.4.0] hexadeca-1(12),13,15-triene-3,11-dione | Esters | Pubchem |
| 81^a^ | 13.29 | [M+H]^+^ 373.1280 | -0.749 | 358.1039, 343.0813, 312.0991 | C20H20O7 | Sinensetin | Flavonoids | (Wang et al., 2025) |
| 82^b^ | 13.48 | [M+H]^+^ 203.1795 | 0.013 | 203.1796, 147.1169, 95.0860 | C15H22 | Caryophyllene epoxide | Terpenes | (Dalvie et al., 2016) |
| 83 | 13.66 | [M+H]^+^ 105.0703 | 3.932 | 105.0703, 87.0448, 59.0693 | C8H8 | Styrene | Others | Pubchem |
| 84 | 13.91 | [M+H]^+^ 233.1536 | -0.070 | 215.1432, 151.1136, 145.1013 | C15H20O2 | Alantolactone | Terpenes | (Qi et al., 2024) |
| 85^a^ | 13.99 | [M-H]^-^  265.1446 | 4.467 | 265.1445, 211.1543, 203.1434 | C_15_H_22_O_4_ | (4aR,5S)-9,9a-dihydroxy-3,4a,5-trimethyl-5,6,7,8,8a,9-hexahydro-4H-benzo[f]benzofuran-2-one | Others | - |
| 86 | 14.03 | [M+H]^+^ 217.1587 | 0.084 | 199.1482, 189.1639, 175.1119, 119.0859 | C15H20O | (+)-ar-Turmerone | Terpenes | (Rasheed et al., 2023) |
| 87 | 14.31 | [M+H]^+^ 403.1385 | -0.556 | 388.1155, 373.0919, 355.0812, 327.0863 | C21H22O8 | Nobiletin | Flavonoids | (Wang et al., 2025) |
| 88 | 14.75 | [M+H]^+^ 223.1692 | -0.164 | 223.1694, 205.1589, 187.1481, 163.1118,  151.1118, 145.1012 | C14H22O2 | Oxyphyllenone H or isomer | Terpenes | (Zhang, 2020) |

Supplementary Table 1 (Continued)

| Peak  NO. | *t*_R_ (min) | Observed Mass | Error (ppm) | MS/MS Fragments | Molecular  Formula | Proposed Compound | Type | Reference/  database |
| --- | --- | --- | --- | --- | --- | --- | --- | --- |
| 89 ^b^ | 14.91 | [M+H]^+^ 235.1692 | -0.283 | 217.1591, 207.1745, 189.1639 | C15H22O2 | Oxyphyllol B | Terpenes | (Shi et al., 2014) |
| 90 | 15.06 | [M+H]^+^ 233.1537 | 0.273 | 233.1538, 215.1432, 205.1581, 191.1069 | C15H20O2 | Eremophila-1(10),11  (12)-dien-2,9-dione | Terpenes | (Zhang, 2020) |
| 91^a^ | 15.19 | [M+NH_4_]^+^ 270.2062 | -0.704 | 235.1694, 217.1591, 199.1483, 189.1638, 133.1014 | C15H24O3 | 2-(8-Hydroxy-4a,8-dimethyldecahydro-2-naph-  thalenyl) acrylic acid | Organic acids | Pubchem |
| 92 | 15.22 | [M+H]^+^ 153.1275 | 0.903 | 136.0759, 135.0808, 107.0860, 93.0704, 91.0550, 79.0459 | C10H16O | Perillyl alcohol | Terpenes | (Qi et al., 2024) |
| 93 | 15.53 | [M+NH_4_]^+^ 270.2062 | -0.482 | 235.1693, 217.1590, 189.1639, 133.1012 | C15H24O3 | Ilicic acid | Terpenes | Pubchem |
| 94^b^ | 16.81 | [M+H]^+^ 221.1901 | 0.308 | 221.1901, 203.1797, 177.1636, 163.1485 | C15H24O | Nootkatol | Terpenes | (Zhang, 2020) |
| 95 | 16.85 | [M-H]^-^  253.0505 | 0.975 | 225.0554, 209.0606, 181.0650, 165.0703 | C15H10O4 | Chrysin | Flavonoids | * |
| 96^b^ | 18.06 | [M+H]^+^ 235.1691 | -0.538 | 235.1691, 217.1590 | C15H22O2 | Confertifolin | Terpenes | (Qi et al., 2024) |
| 97^ab^ | 18.40 | [M+H]^+^ 219.1743 | -0.237 | 219.1745, 191.1793, 177.1277, 163.1119, 151.1119, 149.0963, 137.0962 | C15H22O | Nootkatone | Terpenes | * |
| 98 | 18.53 | [M+H]^+^ 135.0805 | 0.507 | 135.0806, 107.0860, 91.0549 | C9H10O | Chavicol | Organic acids | (Qi et al., 2024) |
| 99^a^ | 18.53 | [M+H-2H2O]^+^ 191.1794 | 0.014 | 191.1795, 135.1170, 107.0860, 95.0861 | C14H26O2 | 2,4,7,9-Tetramethyl-5-decyne-4,7-diol | Others | - |
| 100^ab^ | 19.03 | [M+H]^+^ 203.1795 | 0.456 | 203.1797, 147.1170, 95.0861 | C15H22 | (-)-*α*-Curcumene or isomer | Terpenes | (Qi et al., 2024) |
| 101 | 19.44 | [M+H]^+^ 269.0808 | -0.986 | 269.0811, 254.0574, 226.0625 | C16H12O4 | Tectochrysin | Flavonoids | * |
| 102^ab^ | 19.82 | [M+H]^+^ 219.1744 | 0.037 | 201.1642, 163.1119, 111.0808, 109.1016 | C15H22O | *α*-Cyperone | Terpenes | * |
| 103 | 20.36 | [M+H]^+^ 205.195 | -0.182 | 161.1327, 147.1170, 119.0859, 105.0704 | C15H24 | *β*-Cubebene | Terpenes | (Qi et al., 2024) |
| 104^a^ | 20.74 | [M-H2O+H]^+^ 203.1795 | 0.358 | 203.1797, 147.1170, 105.0703, 95.0861 | C15H24O | Alismol | Terpenes | - |

Supplementary Table 1 (Continued)

| Peak  NO. | *t*_R_ (min) | Observed Mass | Error (ppm) | MS/MS Fragments | Molecular  Formula | Proposed Compound | Type | Reference/  database |
| --- | --- | --- | --- | --- | --- | --- | --- | --- |
| 105 | 21.28 | [M+H]^+^ 293.2109 | -0.721 | 275.2006, 215.1434, 293.2110, 257.1901, 233.1910 | C_18_H_28_O_3_ | 4-hydroxy-6-[2-(2-methyl-1,2,4a,5,6,7,8,8a-octahydronaphthalen-1-yl) ethyl] oxan-2-one | Esters | Pubchem |
| 106 | 21.82 | [M+H]^+^ 205.195 | -0.182 | 205.1963, 163.1484, 149.1328, 135.1170, 121.1015, 107.0860 | C15H24 | Valencene | Terpenes | (Zhang, 2020) |
| 107^a^ | 23.24 | [M+H]^+^ 282.279 | -0.536 | 282.2792, 97.1017, 83.0862, 69.0707 | C18H35NO | Oleamide | Others | - |
| 108^a^ | 25.09 | [M+H]^+^ 256.2634 | -0.356 | 256.2636, 102.0918, 88.0763, 57.0708 | C16H33NO | Palmitamide | Alkaloid | - |

Note: *t*_R_: retention time; * Identified by comparison with reference standards; ^a^ Anotated by GNPS; ^b^ matched with custom-built database in NAP.

**Supplementary Table 2.** **Identification of prototypes of AOF in different biological samples by UPLC-HRMS.**

| Peak NO. | Proposed Compound | SGJ | MB-W | MB-F | FVB | AA | CSF | BT |
| --- | --- | --- | --- | --- | --- | --- | --- | --- |
| 3 | Citric acid | + | - | - | - | - | - | - |
| 6 | Nicotinic acid | + | - | - | - | - | - | - |
| 7 | D-Fructose | + | - | - | - | - | - | - |
| 10 | 2-Aminooctanedioic acid | + | - | - | - | - | - | - |
| 11 | Metharbital | + | - | - | - | - | - | - |
| 14 | 5-Hydroxymethylfurfural | + | + | + | + | + | + | + |
| 15 | Vanillic acid | + | - | - | - | - | - | - |
| 16 | Kojic acid | + | - | - | - | - | - | - |
| 18 | tryptophan N-glucoside | + | - | - | - | - | - | - |
| 19 | Hexaethyleneglycol | + | - | + | - | + | - | - |
| 21 | Protocatechuic acid | + | - | - | - | - | - | - |
| 22 | 6-Hydroxynicotinic acid | + | - | - | - | - | - | - |
| 23 | 3,4-Dihydroxybenzaldehyde | + | + | + | - | + | - | - |
| 24 | Jasmine lactone | + | + | + | + | + | + | + |
| 25 | Oxyphyllenone A | + | + | + | - | + | - | - |
| 26 | Tetrahydroharman-3-carboxylic acid | + | - | - | - | - | - | - |
| 27 | Indoleacetylaspartate | + | - | - | - | - | - | - |
| 28 | Arbutin | + | - | - | - | - | - | - |
| 29 | Isovanillin or isomer | + | - | - | - | + | - | - |
| 30 | Oxyphyllanene A | + | + | + | + | + | + | + |
| 31 | Oxyphyllanene B | + | + | - | - | + | - | - |
| 32 | 3-carboxy-4-methyl-5-propyl-2-Furanpropionic acid | + | - | - | - | + | - | - |
| 33 | Azelaic acid | + | - | - | - | - | - | - |
| 34 | Procyanidin B1 | + | - | - | - | - | - | - |
| 35 | (+)-Catechin | + | - | - | - | - | - | - |
| 36 | Perillic acid | + | + | - | - | - | - | - |
| 37 | Oxyphyllenone B | + | + | + | + | + | + | + |
| 38 | Isovanillin or isomer | + | - | - | - | - | - | - |
| 39 | Oxyphyllone E | + | + | + | + | + | + | + |
| 40 | Verrucarol | + | + | + | + | + | + | - |
| 41 | Oxyphyllenodiol A | + | + | + | + | + | + | - |
| 42 | 1-Carboxycyclohexaneacetic Acid | + | - | - | - | - | - | - |
| 43 | Eugenol | + | - | - | - | - | - | - |
| 44 | Caffeate | + | - | + | - | + | - | - |
| 45 | Monobutyl phthalate or isomer | + | + | + | + | + | + | + |
| 46 | (11S)-Nootkatone-11,12-diol | + | + | + | + | + | - | - |
| 47 | Artemisinin or isomer | + | + | + | - | + | + | - |
| 48 | (11R)-Nootkatone-11,12-diol | + | + | + | + | + | + | + |
| 49 | Senkyunolide H | + | - | + | - | - | - | - |
| 50 | *cis*-4-Coumaric acid | + | - | - | - | - | - | - |
| 51 | *p*-Cymene | + | - | - | - | - | - | - |
| 52 | Teuhetenone A | + | + | + | + | + | + | + |
| 53 | Azelaic acid | + | - | - | - | - | - | - |
| 54 | 8-Deoxylactucin | + | + | + | - | + | - | - |
| 55 | Oxyphyllenodiol B | + | + | + | + | + | + | + |
| 56 | (5S,7R,10R)-5-hydroxy-noreudesma-2-tien-3,11-dione | + | + | + | + | + | - | - |
| 57 | Jasmonic acid | + | - | - | - | - | - | - |
| 58 | Teuhetenone B | + | + | + | + | + | + | + |
| 59 | Oplopanone | + | + | + | + | + | + | + |
| 60 | Oxyphyllone D | + | + | + | + | + | - | - |
| 61 | 7-epi-Teucrenone | + | + | + | + | + | + | + |
| 62 | Artemisinin or isomer | + | + | + | + | + | - | - |
| 63 | (3aR,4R,5aS,6S,9aR,9bS)-4,6-dihydroxy-5a,9-dimethyl-3-methylidene-4,5,6,7,9a,9b-hexahydro-3aH-benzo[g][1] Benzofuran-2-one | + | + | - | + | + | - | - |
| 64 | 2-(8-Hydroxy-4a,8-dimethyldecahydro-2-naph-thalenyl) acrylic acid | + | + | + | + | + | + | + |
| 65 | 2,6-Di-tert-butyl-1,4-benzoquinone | + | + | + | + | + | + | + |
| 66 | Chavicol | + | - | - | - | - | - | - |
| 67 | Costunolide | + | + | - | + | + | - | - |
| 68 | Capsidiol | + | + | + | + | + | + | + |
| 69 | Curcumenol | + | + | + | + | + | - | - |
| 70 | 4-Hexylresorcinol | + | + | + | - | + | - | - |
| 71 | Monobutyl phthalate or isomer | + | - | - | - | - | - | - |
| 72 | Dehydronootkatone | + | + | + | + | + | + | + |
| 73 | 4-Hexylresorcinol isomer | + | - | + | - | + | - | - |
| 74 | (3aR,4R,5aS,6S,9aR,9bS)-4,6-dihydroxy-5a,9-dimethyl-3-methylidene-4,5,6,7,9a,9b-hexahydro-3aH-benzo[g][1] benzo-furan-2-one isomer | + | + | - | - | + | - | - |
| 75 | *β*-asarone | + | - | + | - | - | - | - |
| 76 | Oxyphyllenone H or isomer | + | + | + | + | + | + | + |
| 77 | Parthenolide | + | + | + | + | + | + | + |
| 78 | 9,12,13-Trihydroxyoctadec-10-enoic acid | + | + | + | + | + | - | - |
| 79 | (+) - (4R,5S,7R)-13-Hy-droxynootkatone | + | + | + | + | + | + | + |
| 80 | 5,13,15-Trihydroxy-9-methyl-10-oxabicyclo [10.4.0] hexadeca-1(12),13,15-triene-3,11-dione | + | - | - | - | - | - | - |
| 81 | Sinensetin | + | + | + | + | + | + | + |
| 82 | Caryophyllene epoxide | + | + | + | - | + | - | + |
| 83 | Styrene | - | - | - | - | + | - | - |
| 84 | Alantolactone | + | - | - | - | + | + | - |
| 85 | (4aR,5S)-9,9a-dihydroxy-3,4a,5-trimethyl-5,6,7,8,8a,9-hexahydro-4H-benzo[f]benzofuran-2-one | + | - | - | - | + | - | - |
| 86 | (+)-ar-Turmerone | + | + | + | - | + | - | + |
| 87 | Nobiletin | + | + | + | + | + | + | - |
| 88 | Oxyphyllenone H or isomer | + | + | + | + | + | + | + |
| 89 | Oxyphyllol B | + | + | + | + | + | + | + |
| 90 | Eremophila-1(10),11(12)-dien-2,9-dione | + | - | - | - | + | + | + |
| 91 | 2-(8-Hydroxy-4a,8-dimethyldecahydro-2-naph-thalenyl) acrylic acid | + | - | - | - | + | - | - |
| 92 | Perillyl alcohol | + | - | - | - | + | - | + |
| 93 | Ilicic acid | + | + | + | - | + | - | + |
| 94 | Nootkatol | + | - | - | - | + | - | - |
| 95 | Chrysin | + | - | - | - | - | - | - |
| 96 | Confertifolin | + | + | - | - | + | - | - |
| 97 | Nootkatone | + | + | + | + | + | + | + |
| 98 | Chavicol | + | - | - | - | - | - | - |
| 99 | 2,4,7,9-Tetramethyl-5-decyne-4,7-diol | + | - | - | - | - | - | - |
| 100 | (-)-*α*-Curcumene or isomer | + | - | - | - | - | - | - |
| 101 | Tectochrysin | + | - | - | - | - | - | - |
| 102 | *α*-Cyperone | + | + | + | - | + | + | + |
| 103 | *β*-Cubebene | + | - | - | - | - | - | - |
| 104 | Alismol | + | + | + | - | + | - | - |
| 105 | 4-hydroxy-6-[2-(2-methyl-1,2,4a,5,6,7,8,8a-octahydronaphthalen-1-yl) ethyl]oxan-2-one | + | - | - | - | - | - | - |
| 106 | Valencene | + | - | - | - | - | - | - |

Note: +: Detected; -: Not Detected; SGJ: simulated gastric juice; MB-W: mesenteric blood from intestinal wall metabolism group; MB-F: mesenteric blood from intestinal flora metabolism group; FVB: femoral venous blood from hepatic metabolism; AA: abdominal aorta; CSF: cerebrospinal fluid; BT: brain tissue.

**Supplementary Table 3. Identification of metabolites in different biological samples by UPLC-Q Exactive Orbitrap HRMS**

| Prototypes | NO. | *t*_R_ (min) | Observed Mass | Molecular formula | Error (ppm) | MS/MS Fragments | SGJ | MB-W | MB-F | FVB | AA | CSF | BT |
| --- | --- | --- | --- | --- | --- | --- | --- | --- | --- | --- | --- | --- | --- |
| Teuhetenone A | M_1-1_ | 5.90 | [M+H]^+^  209.1173 | C_12_H_16_O_3_ | 0.283 | 191.1432, 173.1327, 163.1118, 151.1121, 145.1013, 131.0858, 123.0808, 107.0860, 93.0705, 83.0862, 71.0500, 55.0552 | + | - | - | - | + | + | - |
|  | M_1-2_ | 6.12 | [M+H]^+^ 209.1538 | C_13_H_20_O_2_ | 0.686 | 191.1433, 173.1325, 149.1327, 135.0806, 123.0808, 107.0860, 93.0705, 81.0706, 69.0707, 55.0187 | - | + | - | + | + | - | - |
|  | M_1-3_ | 6.80 | [M+H]^+^ 177.1275 | C_12_H_16_O | 0.442 | 159.1171, 149.1327, 131.0858, 121.0651, 107.0496, 93.0705, 81.0706, 69.0707, 55.0551 | - | + | + | + | + | + | + |
|  | M_1-4_ | 7.26 | [M+H]^+^ 181.1223 | C_11_H_16_O_2_ | 0.020 | 163.1120, 145.1014, 135.1171, 123.0808, 121.1015, 107.0861, 93.0705, 81.0706, 71.0500, 55.0186 | + | + | + | + | + | - | - |
| Oxyphylleno-diol B | M_2-1_ | 15.40 | [M+H]^+^ 353.2304 | C_20_H_32_O_5_ | -3.520 | 353.2302, 223.1483, 187.1486, 171.1165, 161.1335, 147.1166, 135.1179, 121.1013, 95.0863, 69.0343, 55.0552 | - | + | - | - | - | - | - |
| Teuhetenone B | M_3-1_ | 2.37 | [M+H]^+^ 250.1438 | C_14_H_19_NO_3_ | 0.160 | 250.1441, 175.1117, 157.1020, 147.1170, 131.09, 119.0859, 105.0705, 91.0550 | + | - | - | - | + | - | - |
|  | M_3-2_ | 3.43 | [M+H]^+^ 252.1598 | C_14_H_21_NO_3_ | 1.348 | 252.1598, 234.1488, 206.1542, 191.1434, 177.1278, 159.1172, 149.1333, 135.1166, 121.1018, 105.0704, 93.0706, 67.0551, 55.0188 | - | + | + | - | - | - | - |

Supplementary Table 3 (Continued)

| Prototypes | NO. | *t*_R_ (min) | Observed Mass | Molecular formula | Error (ppm) | MS/MS Fragments | SGJ | MB-W | MB-F | FVB | AA | CSF | BT |
| --- | --- | --- | --- | --- | --- | --- | --- | --- | --- | --- | --- | --- | --- |
| Teuhetenone B | M_3-3_ | 10.39 | [M+H]^+^ 173.0962 | C_12_H_12_O | 0.742 | 173.1327, 155.0858, 145.1014, 131.0858, 117.0704, 105.0704, 91.0548, 67.0548 | - | - | - | - | + | - | - |
|  | M_3-4_ | 10.42 | [M+H]^+^ 274.1834 | C_14_H_27_NO_2_S | -0.315 | 274.1831, 229.1240, 211.1696, 197.1539, 179.1435, 161.1329, 145.1015, 137.1329, 121.1018, 107.0862, 95.0863, 81.0707, 67.0551, 57.0709 | - | + | + | + | + | - | - |
|  | M_3-5_ | 14.71 | [M+H]^+^ 233.1173 | C_14_H_16_O_3_ | 0.125 | 233.1139, 215.1132, 187.1183, 173.1183, 159.1170, 145.1014, 131.0859, 117.0702, 105.0704, 91.0549, 81.0706, 55.0551 | + | - | - | - | + | - | - |
| Nootkatone | M_4-1_ | 3.15 | [M+H]^+^ 179.1068 | C_11_H_14_O_2_ | 0.523 | 161.0963, 151.1120, 137.0964, 133.1014, 119.0860, 109.1017, 105.0340, 91.0549 | - | - | - | - | + | + | - |
|  | M_4-2_ | 4.90 | [M+H]^+^ 269.1747 | C_15_H_24_O_4_ | -0.096 | 251.1644, 215.1433, 193.1227, 177.1276, 159.1170, 145.1014, 119.0860, 105.0705, 93.0705, 83.0499, 75.0448, 57.0344 | - | + | + | + | + | + | + |
|  | M_4-3_ | 5.16 | [M+H]^+^ 201.1639 | C_15_H_20_ | 0.362 | 183.0654, 173.1327, 159.1170, 145.1013, 131.0857, 119.0859, 107.0860, 93.0705, 81.0706, 69.0707, 55.0551 | - | + | + | + | + | + | + |

Supplementary Table 3 (Continued)

| Prototypes | NO. | *t*_R_ (min) | Observed Mass | Molecular formula | Error (ppm) | MS/MS Fragments | SGJ | MB-W | MB-F | FVB | AA | CSF | BT |
| --- | --- | --- | --- | --- | --- | --- | --- | --- | --- | --- | --- | --- | --- |
| Nootkatone | M_4-4_ | 6.50 | [M+H]^+^ 203.1434 | C_14_H_18_O | 1.763 | 185.1327, 175.1483, 159.1171, 145.1014, 133.1014, 119.0859, 105.0704, 95.0862, 91.0549, 81.0706, 67.0550, 60.0453, 55.0551 | - | + | - | - | - | - | - |
|  | M_4-5_ | 6.80 | [M+H]^+^ 177.1275 | C_12_H_16_O | 0.442 | 159.1171, 149.1327, 131.0858, 121.0651, 107.0496, 93.0705, 81.0706, 69.0707, 55.0551 | + | - | + | + | + | - | + |
|  | M_4-6_ | 6.98 | [M+H]^+^ 163.1118 | C_11_H_14_O | 0.296 | 145.1014, 135.1171, 121.1015, 105.0704, 93.0705, 83.0498, 77.0394, 67.0551, 55.0188, | + | - | - | - | + | - | - |
|  | M_4-7_ | 7.40 | [M+H]^+^ 207.1384 | C_13_H_18_O_2_ | 1.900 | 189.1640, 179.1069, 161.0963, 147.1170, 133.1014, 119.0859, 105.0704, 93.0705, 81.0706, 67.0551 | - | + | - | + | - | - | - |
|  | M_4-8_ | 7.60 | [M+H]^+^ 251.1642 | C_15_H_22_O_3_ | 0.035 | 233.1540, 215.1434, 205.1595, 175.1120, 145.1016, 137.0963, 119.0860, 111.0810, 105.0704, 93.0706, 79.0550 | + | + | + | + | + | + | + |
|  | M_4-9_ | 8.20 | [M+H]^+^ 251.1643 | C_15_H_22_O_3_ | 0.314 | 233.1538, 205.1590, 187.1484, 175.1482, 147.1147, 119.0859, 105.0704, 93.0704, 81.0706 | + | + | + | + | + | + | + |
|  | M_4-10_ | 8.28 | [M+H]^+^ 398.1997 | C_20_H_31_O_5_NS | 0.276 | 380.1891, 356.1890, 293.1570, 267.1415, 217.1590, 177.1276, 159.1170, 130.0501, 84.0451 | - | + | - | + | + | - | - |

Supplementary Table 3 (Continued)

| Prototypes | NO. | *t*_R_ (min) | Observed Mass | Molecular formula | Error (ppm) | MS/MS Fragments | SGJ | MB-W | MB-F | FVB | AA | CSF | BT |
| --- | --- | --- | --- | --- | --- | --- | --- | --- | --- | --- | --- | --- | --- |
| Nootkatone | M_4-11_ | 8.50 | [M+H]^+^ 221.1538 | C_14_H_22_O_2_ | 1.587 | 203.1434, 179.1431, 163.1119, 147.1170, 133.1014, 119.0859, 105.0704, 95.0861, 81.0706, 55.0551 | - | + | - | - | - | - | - |
|  | M_4-12_ | 8.72 | [M+H]^+^ 219.1383 | C_14_H_18_O_2_ | 1.659 | 201.1276, 173.1327, 149.0600, 131.0858, 119.0860, 105.0704, 95.0862, 81.0706, 69.0707, 55.0551 | - | - | - | + | - | - | - |

Note: +: Detected; -: Not Detected; SGJ: simulated gastric juice; MB‐W: mesenteric blood from intestinal wall metabolism group; MB‐F: mesenteric blood from intestinal flora metabolism group; FVB: femoral venous blood from hepatic metabolism; AA: abdominal aorta; CSF: cerebrospinal fluid; BT: brain tissue

Supplementary Table 4 Summary of behavioral, inflammatory, and oxidative stress parameters (mean ± SEM).

| Parameters | Control | Model | SE-AOF-L | SE-AOF-M | SE-AOF-H | Donepezil | Corresponding Figure |
| --- | --- | --- | --- | --- | --- | --- | --- |
| Spontaneous alternation rate (%) | 67.65 ± 4.58 | 39.29 ± 3.80 | 55.46 ± 2.76 | 61.12 ± 5.46 | 63.56 ± 4.85 | 63.84 ± 2.56 | Figure 8D |
| The percentage of entries into the novel arm (%) | 35.25 ± 0.69 | 19.14 ± 3.32 | 27.42 ± 1.40 | 34.70 ± 1.08 | 34.85 ± 1.61 | 33.79 ± 1.96 | Figure 8E |
| The Percentage of time spent in the novel arm (%) | 33.56 ± 1.94 | 12.17 ± 2.64 | 23.65 ± 1.74 | 32.65 ± 5.53 | 31.14 ± 5.73 | 29.77 ± 3.13 | Figure 8F |
| Total distance moved (cm) | 4399.65 ± 323.42 | 1768.22 ± 230.81 | 3088.88 ± 135.16 | 4033.86 ± 368.69 | 3766.59 ± 238.75 | 4165.88 ± 259.71 | Figure 9B |
| Entries into the center area | 46.80 ± 7.45 | 9.80 ± 1.77 | 31.20 ± 4.91 | 47.80 ± 3.85 | 40.20 ± 4.29 | 45.20 ± 5.75 | Figure 9C |
| Time in the center area (s) | 105.60 ± 9.36 | 14.46 ± 3.75 | 52.87 ± 5.56 | 112.69 ± 18.30 | 117.46 ± 9.16 | 119.89 ± 10.46 | Figure 9D |
| TNF-*α* in the plasma (pg/mL) | 54.60 ± 2.74 | 117.93 ± 9.36 | 97.53 ± 11.51 | 73.57 ± 8.53 | 71.20 ± 4.65 | 53.23 ± 7.65 | Figure 11A |
| IL-6 in the plasma (pg/mL) | 16.76 ± 1.34 | 31.76 ± 1.22 | 22.53 ± 1.69 | 18.70 ± 1.67 | 18.45 ± 1.70 | 17.85 ± 1.87 | Figure 11B |
| NO in the brain (μM) | 138.16 ± 4.13 | 323.46 ± 20.61 | 191.16 ± 27.59 | 177.39 ± 24.47 | 201.49 ± 21.11 | 185.26 ± 28.05 | Figure 11C |
| TNF-*α* in the brain (pg/mL) | 186.70 ± 30.29 | 437.30 ± 52.03 | 356.10 ± 11.27 | 270.80 ± 15.42 | 195.13 ± 20.21 | 253.10 ± 34.73 | Figure 11D |
| IL-6 in the brain (pg/mL) | 12.26 ± 0.98 | 23.73 ± 1.60 | 19.33 ± 1.17 | 14.46 ± 1.10 | 14.39 ± 1.00 | 13.45 ± 0.66 | Figure 11E |
| SOD in the plasma (U/mg) | 22.18 ± 0.56 | 15.01 ± 1.91 | 21.39 ± 0.35 | 20.41 ± 0.40 | 21.60 ± 0.84 | 22.03 ± 0.58 | Figure 12A |
| CAT in the plasma (U/mg) | 9.51 ± 0.45 | 4.75 ± 1.00 | 7.91 ± 0.63 | 8.95 ± 0.04 | 10.17 ± 0.71 | 8.04 ± 0.60 | Figure 12B |
| MDA in the plasma (nM/mg) | 6.40 ± 0.23 | 10.70 ± 0.78 | 9.65 ± 0.88 | 8.16 ± 0.26 | 6.05 ± 0.15 | 7.19 ± 0.18 | Figure 12C |
| SOD in the brain (U/mg) | 45.86 ± 2.52 | 31.30 ± 0.99 | 39.99 ± 2.34 | 43.58 ± 1.64 | 43.98 ± 2.37 | 46.09 ± 0.22 | Figure 12D |
| CAT in the brain (U/mg) | 9.95 ± 0.05 | 6.19 ± 1.08 | 10.40 ± 0.28 | 10.92 ± 0.09 | 10.68 ± 0.16 | 10.32 ± 0.61 | Figure 12E |
| MDA in the brain (nM/mg) | 1.97 ± 0.37 | 5.49 ± 0.56 | 3.14 ± 0.73 | 3.04 ± 0.17 | 2.53 ± 0.39 | 3.02 ± 0.35 | Figure 12F |

# References

Dalvie, D. K., Obach, R. S., and Kalgutkar, A. S. (2016). DEALING WITH REALITY: WHEN IS IT NECESSARY TO QUALIFY AND QUANTIFY METABOLITES? SOME CASE STUDIES. *Metabolite Safety in Drug Development*, 261-273.

Enomoto, H., and Miyamoto, K. (2021). Unique localization of jasmonic acid-related compounds in developing Phaseolus vulgaris L. (common bean) seeds revealed through desorption electrospray ionization-mass spectrometry imaging. *Phytochemistry*, 188, 112812. https://doi.org/10.1016/j.phytochem.2021.112812

Inamadugu, J. K., Damaramadugu, R., Mullangi, R., & Ponneri, V. (2010). Simultaneous determination of niacin and its metabolites-nicotinamide, nicotinuric acid and N-methyl-2-pyridone-5-carboxamide-in human plasma by LC-MS/MS and its application to a human pharmacokinetic study. *Biomed Chromatogr,* 24**,** 1059-74. https://doi.org/10.1002/bmc.1406

Jeong, W. T., An, S. J., and Lim, H. B. (2021). Rapid Determination of Furoquinoline Alkaloids in Rutaceae Species by Ultra-Performance Liquid Chromatography (UPLC) with Photodiode Array (PDA) and Electrospray Ionization–Quadrupole Time-of-Flight Mass Spectrometry (ESI-Q-TOF/MS). *Analytical Letters,* 54**,** 698-715.

Lang, R., Yagar, E. F., Eggers, R., and Hofmann, T. (2008). Quantitative investigation of trigonelline, nicotinic acid, and nicotinamide in foods, urine, and plasma by means of LC-MS/MS and stable isotope dilution analysis. *J Agric Food Chem,* 56**,** 11114-21. https://doi.org/10.1021/jf802838s

Patras, M. A., Jaiswal, R., McDougall, G. J., and Kuhnert, N. (2018). Profiling and Quantification of Regioisomeric Caffeoyl Glucoses in Berry Fruits. *J Agric Food Chem,* 66**,** 1096-1104. https://doi.org/10.1021/acs.jafc.7b02446

Qi, Y., Zhou, Q., Zhang, Y., Deng, J., Li, R., and Zhang, X. (2024). Exploring the active components and potential mechanisms of *Alpiniae oxyphyllae* Fructus in treating diabetes mellitus with depression by UPLC-Q-Exactive Orbitrap/MS, network pharmacology and molecular docking. *Metab Brain Dis,* 39**,** 1065-1084. https://doi.org/10.1007/s11011-024-01374-z

Rasheed, D. M., Farag, M. A., Khattab, A. R., Rahman, M. F. A., and El-Haddad, A. E. (2023). A comparative MS-based metabolomics study and in-vitro antidiabetic assay of galangals, turmeric and ginger aided by molecular networking and chemometrics. *Industrial Crops and Products,* 205**,** 117438.

Ruan, X. 2021. *Study of Bioactive Ingredients and Product Developmentof Alpiniae Oxyphyllae Fructus in Guangxi.* master.

Shi, S. H., Zhao, X., Liu, B., Li, H., Liu, A. J., Wu, B., Bi, K. S., and Jia, Y. (2014). The effects of sesquiterpenes-rich extract of *Alpinia oxyphylla* Miq. on amyloid-β-induced cognitive impairment and neuronal abnormalities in the cortex and hippocampus of mice. *Oxid Med Cell Longev,* 2014**,** 451802. https://doi.org/10.1155/2014/451802

Sun, Z., Kong, X., Zuo, L., Kang, J., Hou, L., & Zhang, X. (2016). Rapid extraction and determination of 25 bioactive constituents in *Alpinia oxyphylla* using microwave extraction with ultra high performance liquid chromatography with tandem mass spectrometry. *J Sep Sci,* 39**,** 603-10. https://doi.org/10.1002/jssc.201501056

Von Bargen, K. W., Niehaus, E. M., Bergander, K., Brun, R., Tudzynski, B., and Humpf, H. U. (2013). Structure elucidation and antimalarial activity of apicidin F: an apicidin-like compound produced by Fusarium fujikuroi. *J Nat Prod,* 76**,** 2136-40. https://doi.org/10.1021/np4006053

Wang, X., Yi, Z., Zhang, Y., Zhang, J., Li, X., Qi, D., et al (2025). Identification and Therapeutic Potential of Polymethoxylated Flavones in *Citri Reticulatae* Pericarpium for Alzheimer's Disease: Targeting Neuroinflammation. *Molecules,* 30(4), 771. https://doi.org/10.3390/molecules30040771

Wu, Q., Ou, C., Wang, J., Wu, X., Gao, Z., Zhao, Y., et al (2024) Jiawei Kongsheng Zhenzhong Pill: marker compounds, absorption into the serum (rat), and Q-markers identified by UPLC-Q-TOF-MS/MS. *Front Pharmacol,* 15**,** 1328632. https://doi.org/10.3389/fphar.2024.1328632

Zhang, M. 2020. *Pharmacodynamics and Mechanical Study of AlpiniaeOxyphyllae Fructus in the Treatment ofAlzheimer's DiseaseBased on Metabolomics and Network Pharmacology.* master, The First Affiliated Hospital of Zhengzhou University.
