# Supplementary figures and images for: Identification, biotransformation, and neuroprotective potential of the ethanol extract of Alpiniae oxyphyllae fructus in neuroinflammation-related cognitive impairment
Source: Front Pharmacol. 2025 Nov 26;16:1714500. doi: 10.3389/fphar.2025.1714500 (PMC12689551; doi:10.3389/fphar.2025.1714500)

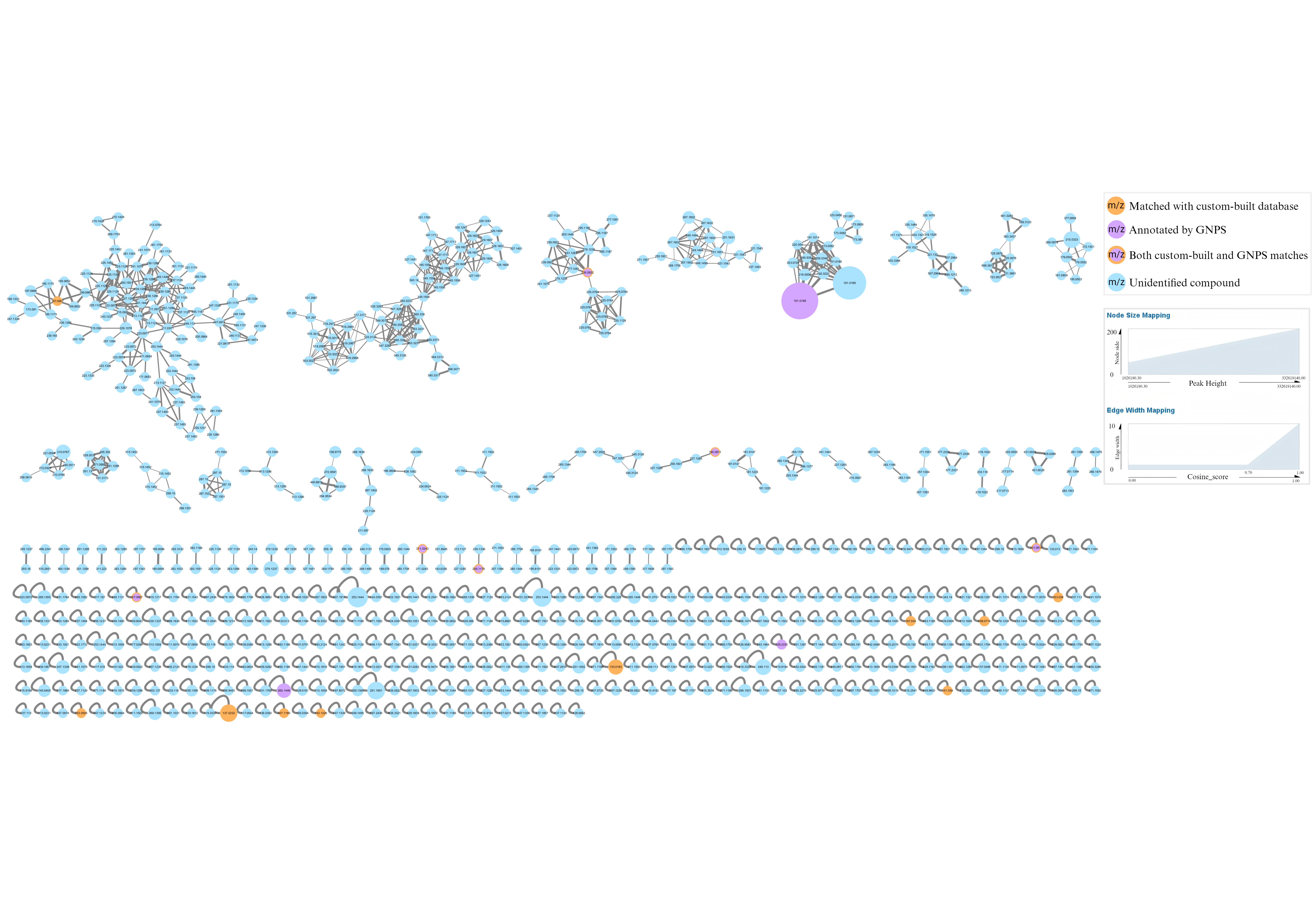

Supplement: Supplementary file 1 [file DataSheet1.zip › Supplementary Material/Figure S1.jpeg]

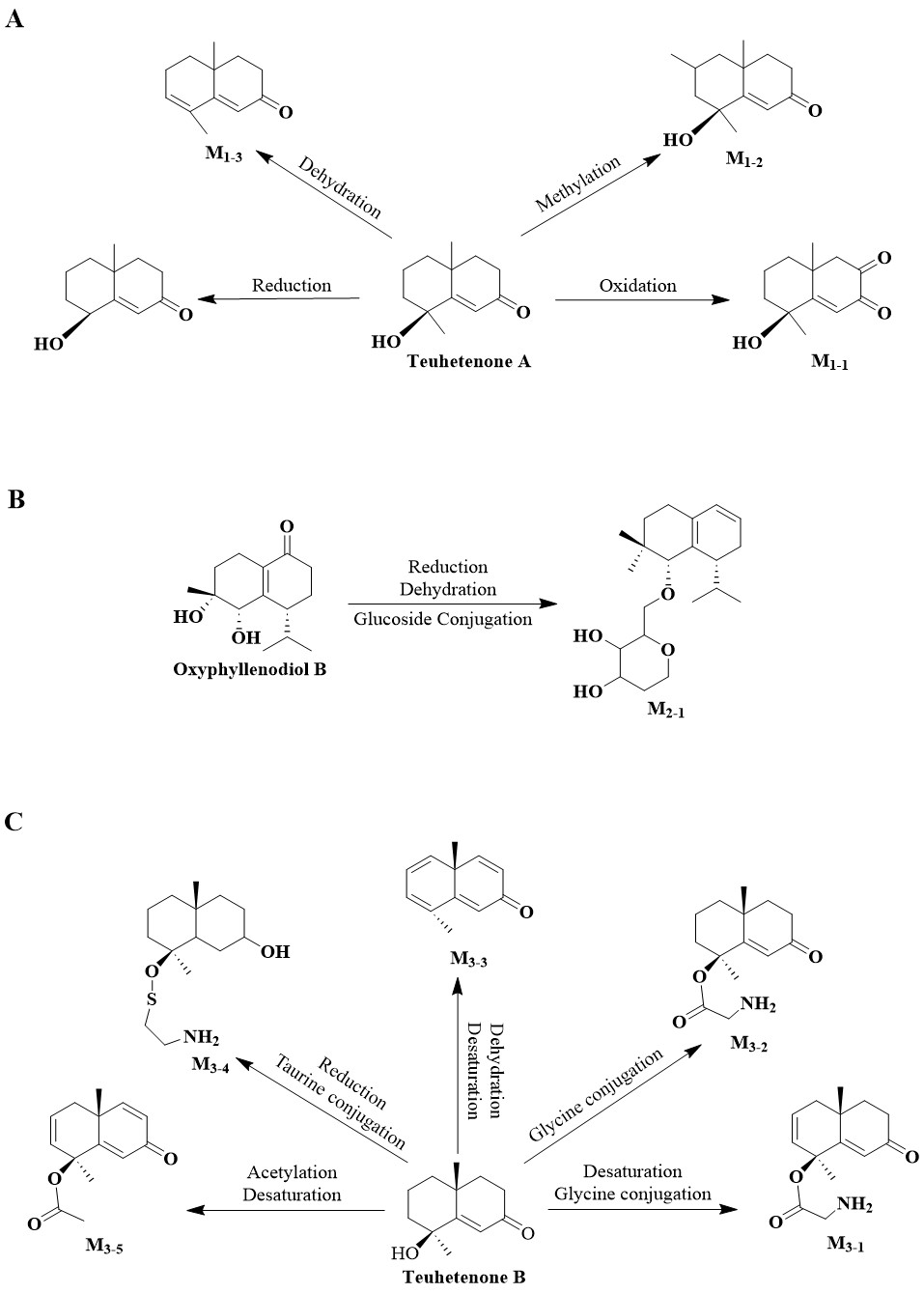

Supplement: Supplementary file 1 [file DataSheet1.zip › Supplementary Material/Figure S3.jpg]

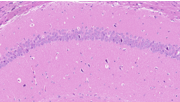

Supplement: Supplementary file 1 [file DataSheet1.zip › Supplementary Material/Microscopy images/Figure 10/Figure 10A/A-1-Control-Hippocampus CA1-40μm.tif]

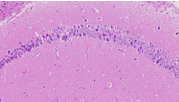

Supplement: Supplementary file 1 [file DataSheet1.zip › Supplementary Material/Microscopy images/Figure 10/Figure 10A/A-2-Model-Hippocampus CA1-40μm.tif]

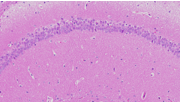

Supplement: Supplementary file 1 [file DataSheet1.zip › Supplementary Material/Microscopy images/Figure 10/Figure 10A/A-3-SE-AOF-L-Hippocampus CA1-40μm.tif]

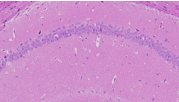

Supplement: Supplementary file 1 [file DataSheet1.zip › Supplementary Material/Microscopy images/Figure 10/Figure 10A/A-4-SE-AOF-M-Hippocampus CA1-40μm.tif]

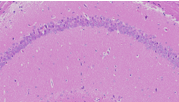

Supplement: Supplementary file 1 [file DataSheet1.zip › Supplementary Material/Microscopy images/Figure 10/Figure 10A/A-5-SE-AOF-H-Hippocampus CA1-40μm.tif]

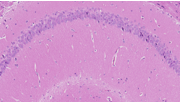

Supplement: Supplementary file 1 [file DataSheet1.zip › Supplementary Material/Microscopy images/Figure 10/Figure 10A/A-6-Donepezil-Hippocampus CA1-40μm.tif]

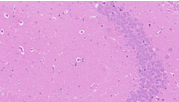

Supplement: Supplementary file 1 [file DataSheet1.zip › Supplementary Material/Microscopy images/Figure 10/Figure 10B/B-1-Control-Hippocampus CA3-40μm.tif]

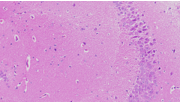

Supplement: Supplementary file 1 [file DataSheet1.zip › Supplementary Material/Microscopy images/Figure 10/Figure 10B/B-2-Model-Hippocampus CA3-40μm.tif]

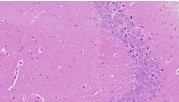

Supplement: Supplementary file 1 [file DataSheet1.zip › Supplementary Material/Microscopy images/Figure 10/Figure 10B/B-3-SE-AOF-L-Hippocampus CA3-40μm.tif]

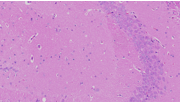

Supplement: Supplementary file 1 [file DataSheet1.zip › Supplementary Material/Microscopy images/Figure 10/Figure 10B/B-4-SE-AOF-M-Hippocampus CA3-40μm.tif]

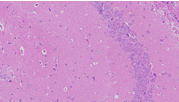

Supplement: Supplementary file 1 [file DataSheet1.zip › Supplementary Material/Microscopy images/Figure 10/Figure 10B/B-5-SE-AOF-H-Hippocampus CA3-40μm.tif]

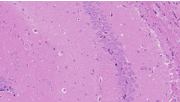

Supplement: Supplementary file 1 [file DataSheet1.zip › Supplementary Material/Microscopy images/Figure 10/Figure 10B/B-6-Donepezil-Hippocampus CA3-40μm.tif]

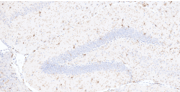

Supplement: Supplementary file 1 [file DataSheet1.zip › Supplementary Material/Microscopy images/Figure 10/Figure 10C/C-1-Control-Hippocampus-100μm.tif]

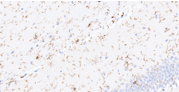

Supplement: Supplementary file 1 [file DataSheet1.zip › Supplementary Material/Microscopy images/Figure 10/Figure 10C/C-1-Control-Hippocampus-50μm.tif]

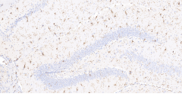

Supplement: Supplementary file 1 [file DataSheet1.zip › Supplementary Material/Microscopy images/Figure 10/Figure 10C/C-2-Model-Hippocampus-100μm.tif]

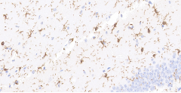

Supplement: Supplementary file 1 [file DataSheet1.zip › Supplementary Material/Microscopy images/Figure 10/Figure 10C/C-2-Model-Hippocampus-50μm.tif]

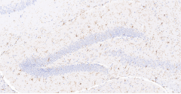

Supplement: Supplementary file 1 [file DataSheet1.zip › Supplementary Material/Microscopy images/Figure 10/Figure 10C/C-3-SE-AOF-L-Hippocampus-100μm.tif]

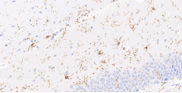

Supplement: Supplementary file 1 [file DataSheet1.zip › Supplementary Material/Microscopy images/Figure 10/Figure 10C/C-3-SE-AOF-L-Hippocampus-50μm.tif]

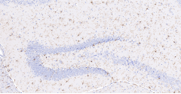

Supplement: Supplementary file 1 [file DataSheet1.zip › Supplementary Material/Microscopy images/Figure 10/Figure 10C/C-4-SE-AOF-M-Hippocampus-100μm.tif]

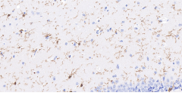

Supplement: Supplementary file 1 [file DataSheet1.zip › Supplementary Material/Microscopy images/Figure 10/Figure 10C/C-4-SE-AOF-M-Hippocampus-50μm.tif]

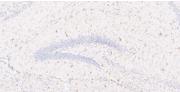

Supplement: Supplementary file 1 [file DataSheet1.zip › Supplementary Material/Microscopy images/Figure 10/Figure 10C/C-5-SE-AOF-H-Hippocampus-100μm.tif]

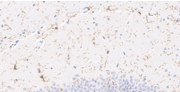

Supplement: Supplementary file 1 [file DataSheet1.zip › Supplementary Material/Microscopy images/Figure 10/Figure 10C/C-5-SE-AOF-H-Hippocampus-50μm.tif]

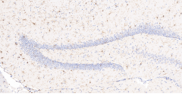

Supplement: Supplementary file 1 [file DataSheet1.zip › Supplementary Material/Microscopy images/Figure 10/Figure 10C/C-6-Donepezil-Hippocampus-100μm.tif]

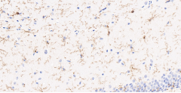

Supplement: Supplementary file 1 [file DataSheet1.zip › Supplementary Material/Microscopy images/Figure 10/Figure 10C/C-6-Donepezil-Hippocampus-50μm.tif]

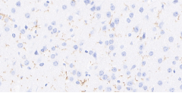

Supplement: Supplementary file 1 [file DataSheet1.zip › Supplementary Material/Microscopy images/Figure 10/Figure 10C/D-1-Control-Cortical-20μm.tif]

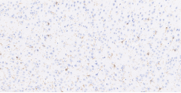

Supplement: Supplementary file 1 [file DataSheet1.zip › Supplementary Material/Microscopy images/Figure 10/Figure 10D/D-1-Control-Cortical-50μm.tif]

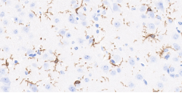

Supplement: Supplementary file 1 [file DataSheet1.zip › Supplementary Material/Microscopy images/Figure 10/Figure 10D/D-2-Model-Cortical-20μm.tif]

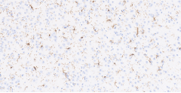

Supplement: Supplementary file 1 [file DataSheet1.zip › Supplementary Material/Microscopy images/Figure 10/Figure 10D/D-2-Model-Cortical-50μm.tif]

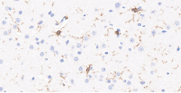

Supplement: Supplementary file 1 [file DataSheet1.zip › Supplementary Material/Microscopy images/Figure 10/Figure 10D/D-3-SE-AOF-L-Cortical-20μm.tif]

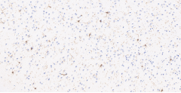

Supplement: Supplementary file 1 [file DataSheet1.zip › Supplementary Material/Microscopy images/Figure 10/Figure 10D/D-3-SE-AOF-L-Cortical-50μm.tif]

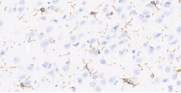

Supplement: Supplementary file 1 [file DataSheet1.zip › Supplementary Material/Microscopy images/Figure 10/Figure 10D/D-4-SE-AOF-M-Cortical-20μm.tif]

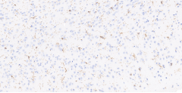

Supplement: Supplementary file 1 [file DataSheet1.zip › Supplementary Material/Microscopy images/Figure 10/Figure 10D/D-4-SE-AOF-M-Cortical-50μm.tif]

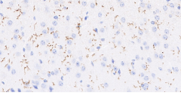

Supplement: Supplementary file 1 [file DataSheet1.zip › Supplementary Material/Microscopy images/Figure 10/Figure 10D/D-5-SE-AOF-H-Cortical-20μm.tif]

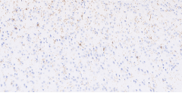

Supplement: Supplementary file 1 [file DataSheet1.zip › Supplementary Material/Microscopy images/Figure 10/Figure 10D/D-5-SE-AOF-H-Cortical-50μm.tif]

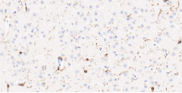

Supplement: Supplementary file 1 [file DataSheet1.zip › Supplementary Material/Microscopy images/Figure 10/Figure 10D/D-6-Donepezil-Cortical-20μm.tif]

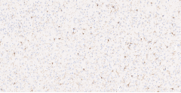

Supplement: Supplementary file 1 [file DataSheet1.zip › Supplementary Material/Microscopy images/Figure 10/Figure 10D/D-6-Donepezil-Cortical-50μm.tif]
